# The Not5 Subunit of the Ccr4-Not Complex Connects Transcription and Translation

**DOI:** 10.1371/journal.pgen.1004569

**Published:** 2014-10-23

**Authors:** Zoltan Villanyi, Virginie Ribaud, Sari Kassem, Olesya O. Panasenko, Zoltan Pahi, Ishaan Gupta, Lars Steinmetz, Imre Boros, Martine A. Collart

**Affiliations:** 1 Department of Microbiology and Molecular Medicine, University of Geneva, Faculty of Medicine, Geneva, Switzerland; 2 Institute of Genetics and Genomics of Geneva, Geneva, Switzerland; 3 Department of Biochemistry and Molecular Biology, University of Szeged, Szeged, Hungary; 4 European Molecular Biology Laboratory (EMBL), Genome Biology Unit, Heidelberg, Germany; Technion, Israel Institute of Technology, Israel

## Abstract

Recent studies have suggested that a sub-complex of RNA polymerase II composed of Rpb4 and Rpb7 couples the nuclear and cytoplasmic stages of gene expression by associating with newly made mRNAs in the nucleus, and contributing to their translation and degradation in the cytoplasm. Here we show by yeast two hybrid and co-immunoprecipitation experiments, followed by ribosome fractionation and fluorescent microscopy, that a subunit of the Ccr4-Not complex, Not5, is essential in the nucleus for the cytoplasmic functions of Rpb4. Not5 interacts with Rpb4; it is required for the presence of Rpb4 in polysomes, for interaction of Rpb4 with the translation initiation factor eIF3 and for association of Rpb4 with mRNAs. We find that Rpb7 presence in the cytoplasm and polysomes is much less significant than that of Rpb4, and that it does not depend upon Not5. Hence Not5-dependence unlinks the cytoplasmic functions of Rpb4 and Rpb7. We additionally determine with RNA immunoprecipitation and native gel analysis that Not5 is needed in the cytoplasm for the co-translational assembly of RNA polymerase II. This stems from the importance of Not5 for the association of the R2TP Hsp90 co-chaperone with polysomes translating *RPB1* mRNA to protect newly synthesized Rpb1 from aggregation. Hence taken together our results show that Not5 interconnects translation and transcription.

## Author Summary

In this work we show that, both in the nucleus and in the cytoplasm, Not5 plays a “bridging” role for RNA Polymerase II. In the cytoplasm, Not5 interacts with the mRNA encoding the largest subunit of RNA polymerase II Rpb1 and supports the association of a co-chaperone to newly produced protein, to keep it soluble and assembly competent. In the nucleus, Not5 interacts with the Rpb4 subunit of polymerase that is known to readily dissociate from the rest of the polymerase, and it is essential for Rpb4 to associate with mRNAs at the completion of transcription to contribute to translation and mRNA degradation in the cytoplasm. Hence our data define Not5 as a key player in the cross-talk between different stages of eukaryotic gene expression: Not5 impacts on production of polymerase, hence transcription, during translation, and on Rpb4 mRNA association, hence translation and mRNA degradation during transcription.

## Introduction

The life of an mRNA molecule in eukaryotic cells is considered to be the sum of distinct events separated in time and space. Precisely, this separation seems to constitute the characteristic difference distinguishing eukaryotes from prokaryotes, where translation is co-transcriptional and occurs in a single cellular compartment. Several studies in recent years, however, have challenged this simple view. First, the heptapeptide repeat-containing C-terminal domain (CTD) of the largest subunit of eukaryotic RNA polymerase II (RNA Pol II) was found to direct post-transcriptional RNA processing events. It serves as a landing platform for components of the machines involved in mRNA capping, splicing, and mRNA export [1], [2], [3]. More recently and provocatively, an RNA Pol II subunit, Rpb4, has been suggested to play roles not only in the nucleus during the transcription process, but also subsequently in the cytoplasm, contributing to both the RNA degradation and translation processes [4], [5].

The conserved eukaryotic Ccr4-Not complex also contributes to both transcription and mRNA decay and is found both in the cytoplasm and nucleus (for reviews see [6], [7]). The complex consists of 9 subunits in the yeast *Saccharomyces cerevisiae* (Ccr4, Caf1, Caf40, Caf130, and Not1-5). The single CNot3 protein of higher eukaryotes, whether human [8] or fly [9] corresponds to yeast Not3 and Not5, which share 44% identity in their N-termini. In these eukaryotes, the complex also carries additional subunits CNot10 and CNot11, and lacks Caf130 [10], [11], [12], [13]. The Ccr4-Not complex plays roles at several stages of gene expression. Many subunits of Ccr4-Not can be cross-linked to genes being transcribed [14], [15], [16], the complex interacts with RNA Pol II and contributes to transcription elongation [17], and the Not subunits impact on the distribution of general transcription initiation factors across the genome [14], [18]. The Ccr4 and Caf1 subunits comprise the major eukaryotic deadenylase and catalyze the first and rate-limiting step of RNA degradation ([19] and for review see [20]). Recent studies in yeast have established that some subunits of the Ccr4-Not complex are present at translating ribosomes (polysomes) [21], [22] and that the level of polysomes is reduced in certain Ccr4-Not deletion mutants. This coincides with an accumulation of aggregated proteins in the mutants [21], [23], and with the importance of the Ccr4-Not complex for the assembly of the multi-subunit proteasome complex [24].

The functional implication of both the Ccr4-Not complex and the Rpb4 subunit of RNA Pol II to all stages of the mRNA life cycle was supported by a recent study revealing that transcription and mRNA degradation rates have co-evolved oppositely and that this coincides with single nucleotide changes in either *RPB4* or *CCR4-NOT* genes [25]. These studies indicate that Rpb4, which connects transcription to downstream events, may somehow connect the polymerase also to the Ccr4-Not complex. Intriguingly however, it has been reported that the interaction of polymerase with the Ccr4-Not complex, does not require Rpb4 [17].

RNA Pol II consists of 12 subunits, 10 of which compose the catalytic core. These are shared with RNA Pol I and RNA Pol III or related to subunits of these other polymerases [26]. Several recent studies have provided insight into the mechanism by which the 12-subunit RNA Pol II is assembled (reviewed in [27]). The finding that partially assembled polymerase complexes accumulated in the cytoplasm under conditions of imbalanced levels of different RNA Pol II subunits suggested cytoplasmic assembly of this enzyme. For instance, treatment of cells with α-amanitin leads to specific degradation of Rpb1 from elongation-stalled polymerase and to the accumulation of a cytoplasmic Rpb2 sub-complex containing Rpb3, Rpb10, Rbp11 and Rpb12 [28]. In contrast, inhibition of the *de-novo* synthesis of any Pol II subunit besides Rpb1 by siRNA leads to the accumulation of cytoplasmic Rpb1. This pool of Rpb1 is mostly unphosphorylated and insensitive to α-amanitin suggesting that it is newly synthesized Rbp1, which has not been engaged in transcription. The cytoplasmic assembly complexes have been characterized by mass-spectrometry-based proteomics [28] and found to represent two intermediates. The Rpb2 sub-complex contains the Gpn1/Npa3, Gpn2 and Gpn3 GTP binding proteins and several chaperones. Another sub-complex contains Rpb1 with the Hsp90 chaperone and its R2TP co-chaperone. In yeast, R2TP is composed of the Tah1 tetratricopeptide repeat (TPR) protein, Pih1 (protein interacting with Hsp90) and the two AAA+ ATPases, Rvb1 and Rvb2. Pih1 binds Tah1, the Rvb proteins and the yeast Hsp90 chaperones Hsp82 and/or Hsc82 [29], [30]. The two RNA Pol II assembly intermediates join, and then enter the nucleus mainly via the nuclear transport of fully assembled polymerase [28]. The transport requires association of the assembled polymerase with an NLS-containing protein Iwr1 [31]. GTP binding might also play a role in assembly and/or nuclear import of assembled RNA Pol II, since depletion of the human GTP binding protein Gpn1, or mutation of its yeast ortholog Npa3, leads to cytoplasmic accumulation of polymerase subunits [32], [33]. Another protein recently identified as playing a role in assembly of RNA Pol II and also RNA Pol I and RNA Pol III is the Bud27 prefoldin [34]. It is required for the correct integration of Rpb5 and Rpb6 into all three polymerases and acts prior to nuclear import.

Rpb4 forms a hetero-dimeric sub-complex with Rpb7, which extends like a stalk from the core of RNA Pol II. The Rpb4/7 dimer can dissociate from the rest of RNA Pol II and is in excess to the other RNA polymerase II subunits. It shuttles between the cytoplasm and the nucleus, interacts with the translation scaffold factor eIF3 and is required for wild-type levels of translating polysomes [4], [35], [36] and for review see [37]. Curiously, while Rpb4 plays an essential role in connecting transcription to downstream steps in gene expression, it is not essential for yeast viability.

In this work we investigated how Rpb4 and the Ccr4-Not complex are functionally connected. We determined that Rpb4 shows a very tight two-hybrid interaction with the Not5 subunit of the Ccr4-Not complex. In addition, we found that the presence of Rpb4 in translating ribosomes, and more globally the association of Rpb4 with mRNAs, was dependent upon nuclear Not5. We observed that not only Rpb4, but also several other RNA Pol II subunits were present in polysomes. These findings are consistent with cytoplasmic assembly of RNA Pol II occurring on translating ribosomes, as suggested for protein complexes quite generally [38]. Moreover our data indicates that cytoplasmic Not5 contributes to RNA Pol II assembly at least in part by supporting interaction of *de-novo* synthesized Rpb1 with Hsp90-R2TP co-chaperone and preserving a soluble pool of Rpb1 that is apt to interact with Rpb2 to form new RNA Pol II complexes. We determined that this role of Not5 for Rpb1 solubility is conserved in *Drosophila melanogaster*. Hence, Not5 is in a central position for the bidirectional communication between transcription and translation.

## Results

### Not5 is required for the cytoplasmic functions of Rpb4

**Figure 1:**
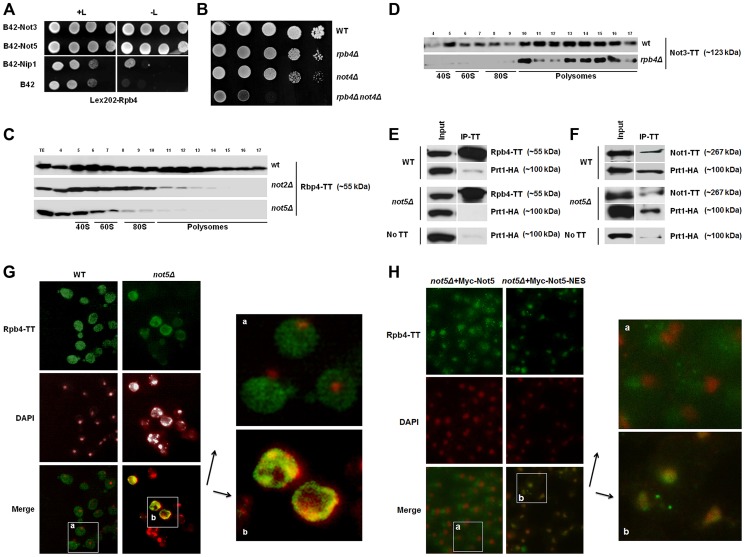
Rpb4 interacts with Not5 and the cytoplasmic functions of Rpb4 require Not5. **A**. Serial dilutions of exponentially growing reporter cells expressing LexA-Rpb4 as a bait, and the indicated proteins fused to B42 as preys, were spotted either on medium selective for the plasmids (left panel +L) or selective for the plasmids and indicative of an interaction between bait and prey (right panel -L). **B**. Serial dilutions of exponentially growing cells from the indicated strains were spotted on plates and left to grow for several days at 30°C. **C** and **D**. Fractions from 7–47% sucrose gradients of extracts from wild-type or mutant strains expressing the indicated Tap-tagged (TT) proteins were precipitated with TCA and analyzed by western blotting with PAP antibodies. The positions of 40S, 60S, 80S and polysomes are indicated under the blots. The numbers of the gradient fractions tested or the total extract (TE) are indicated at the top. The polysome profiles for these experiments are available in Fig. S15 along with a typical distribution of a ribosomal protein (Rps3) in the wt and *not5Δ* gradients. Rpb4-TT (**E**) or Not1-TT (**F**) were immunoprecipitated from extracts of wild-type or mutant cells expressing HA-tagged Prt1. Wild-type cells expressing untagged Rpb4 or Not1 were used as a control. Similar negative controls were obtained with *not5Δ* cells not expressing any Tap-tagged protein (Fig. S16). The immunoblots were developed using anti-CBP or HA antibodies. **G** and **H**. Wild-type and *not5Δ* cells expressing Rpb4-TT (**G**) or the indicated (**H**) Not5 derivatives, were grown exponentially and stained with anti-CBP antibodies (upper panels) or DAPI (middle panels). The pictures were merged (lower panels) and the indicated section from wild-type (a) or *not5Δ* (b) was enlarged for better visualization. The localization of the Not5 derivatives is presented in Fig. S2.

The importance of the Ccr4-Not complex during the entire life of mRNAs (for review see [6]) is reminiscent of the extended function described for the Rpb4 subunit of RNA Pol II [37]. We hence investigated by the two-hybrid assay whether the Ccr4-Not complex interacts with Rpb4. We used Rpb4 as bait and tested its interaction against each of the Ccr4-Not subunits as preys. As positive control we used Nip1, an eIF3 subunit with which Rpb4 has been shown to interact [4]. We observed strong two-hybrid interactions of Rpb4 with both Not3 and Not5. In both cases the detected interaction was more remarkable than that between Rpb4 and its known partner Nip1 (Fig. 1A). A weaker interaction between Rpb4 and the other Ccr4-Not subunits was also evident (Fig. S1A) and they could be confirmed by co-immunoprecipitation experiments (Fig. S1B) even in the presence of RNase indicating that the interaction is not bridged by RNA. Not3 and Not5 share extended sequence homology and may have evolved in yeast from a common ancestral gene, since in higher eukaryotes there is a single gene encoding this subunit of the Ccr4-Not complex (reviewed in [39]). The deletion of either Not3 or Not5 is lethal when combined with the deletion of Not4 [40] so we tested whether this genetic interaction was shared by Rpb4 that is not a subunit of the Ccr4-Not complex, but interacts with both Not3 and Not5. Indeed, the deletion of Rpb4 displayed a striking slow growth phenotype when combined with Not4 (Fig. 1B). A synthetic slow growth phenotype was also detected when *rpb4Δ*, like *not5Δ* [40], was combined with *ccr4Δ*, but not when it was combined with the deletion of Caf40, another Ccr4-Not subunit (Fig. S1C).

Rpb4 has been connected to translation and found in polysomes [4], and this has also been established for certain Ccr4-Not subunits (Not4 and Not5) [21], [22], [23]. This led us to test whether the presence of Rpb4 and Ccr4-Not subunits in polysomes was interdependent. Not2 and Not5 were essential for the detection of Rpb4 in polysomes (Fig. 1C). When trying to do the reverse experiment we were unable to create an *rpb4Δ* strain expressing tagged Not5, probably because of synthetic lethality issues (see below). Hence instead we followed the fractionation of tagged Not3 in cells lacking Rpb4. Not3 was present in polysomes even in cells lacking Rpb4 (Fig. 1D).

Rpb4 was first connected to translation through its interaction with the translation initiation factor eIF3 [4]. We could recapitulate this interaction by co-immunoprecipitating a subunit of eIF3, Prt1, with Rpb4 (Fig. 1E). Since we observed that Rpb4 was not present in polysome fractions in *not2Δ* and *not5Δ*, it was of interest to determine whether Rpb4 could still interact with eIF3 in these mutants. We could not detect co-immunoprecipitation of Prt1 with Rpb4 in *not5Δ* cells, suggesting that the interaction of Rpb4 with eIF3 is dependent upon Not5 (Fig. 1E). Consistent with a role for the Ccr4-Not complex in mediating the interaction of Rbp4 with eIF3, we found that Prt1 co-immunoprecipitates with Not1 (Fig. 1F). In addition two-hybrid experiments revealed interactions between another eIF3 subunit, Nip1 and many Ccr4-Not subunits (Fig. S1D). The Not1-eIF3 interaction was independent of Not5 (Fig. 1F) in good correlation with the observation that Not1 association with polysome fractions does not depend upon Not5 but is dependent upon intact polysomes (Fig. S1E).

Rpb4 has been shown to shuttle between the nucleus and the cytoplasm and accumulates in the cytoplasm under stress conditions that can be mimicked by fixing yeast cells with formaldehyde [41]. Under such conditions, we could confirm a largely cytoplasmic localization of Rpb4 in wild-type cells (Fig. 1G). In contrast, in cells lacking Not5, Rpb4 was present in the cytoplasm in a lesser amount and accumulated in nuclei (Fig. 1G). Moreover, the nuclei tended to display aberrant morphologies when cells lacked Not5 and expressed tagged Rpb4. This synthetic phenotype was consistent with our observation that no viable spores lacking both Not5 and Rpb4 germinated upon dissection of diploids obtained from crossing *rpb4Δ* with *not5Δ* and that attempts to create an *rpb4Δ* strain expressing a C-terminally tagged Not5 was unsuccessful as mentioned above. The expression of a derivative of Not5 maintained in the cytoplasm by a nuclear export signal (NES) in the *not5Δ* strain expressing tagged Rpb4 (Fig S2) could not rescue the stress-induced cytoplasmic localization of Rpb4, in contrast to its wild-type counterpart (Fig. 1H).

The presence of Rpb4 in the cytoplasm was reported to result from its interaction with mRNAs occurring at the completion of transcription [36]. We thus analyzed the interaction of Rpb4 with a couple of mRNAs, namely *RPB1* and *NIP1*, in wild-type cells or in cells lacking Not5. Rpb4 is expressed at similar levels in both strains, and is immunoprecipitated to similar extents in both strains (Fig. S3). *RPB1* mRNA was significantly enriched in the Rpb4 immunoprecipitates from wild-type cells but not from *not5Δ* cells, and in parallel significant binding of Not5 to *RBP1* mRNA was detected (Fig. S4). In contrast, no significant enrichment of *NIP1* mRNA could be detected in the Rpb4 immunoprecipitates from either strain, but it is nevertheless noteworthy that less *NIP1* mRNA was immunoprecipitated with Rpb4 from *not5Δ* than from the wild-type (Fig S4). The relative presence of *NIP1* mRNA in the immunoprecipitate versus total mRNA was similar for Rpb4 and Rpl17, a ribosomal protein expected to be associated with all translated mRNAs (Fig. S4). This indicates that Rpb4 is probably associated with *NIP1* mRNA, but that the representation of *NIP1* mRNA in the pool of mRNAs associated with Rpb4 is not higher than its representation in the pool of total mRNAs. This is in contrast to *RPB1* that is more enriched in the immunoprecipitates of Rpb4 than Rpl17, and significantly enriched in the immunoprecipitate versus total mRNA of Rpb4, but not Rpl17. Neither *RPB1* nor *NIP1* mRNAs were immunoprecipitated from an untagged strain confirming that the immunoprecipitations were specific.

### Many RNA Pol II subunits associate with polysomes

**Figure 2:**
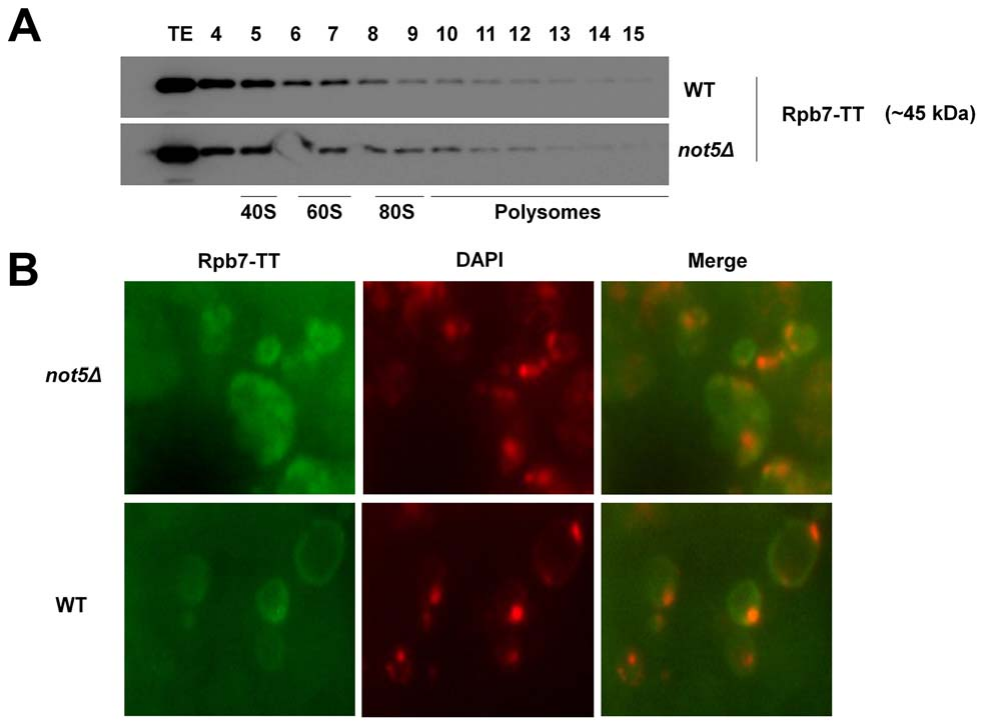
Presence of Rpb7 in polysomes or in the cytoplasm is not affected by Not5. **A**. Wild-type or mutant cells expressing Tap-tagged Rpb7 as indicated were analyzed on sucrose gradients as in Fig. 1C. The polysome profiles and protein loading for these experiments are available in Fig. S15. **B**. Wild-type and *not5Δ* cells expressing Rpb7-TT were grown exponentially and stained with anti-CBP antibodies or DAPI as for Fig. 1G. The merged pictures are displayed.

Previously not only Rpb4, but also its partner protein Rpb7 has been implicated in translation and cytoplasmic mRNA degradation [4], [42]. The Rpb4 and Rpb7 proteins are thought to shuttle between nucleus and cytoplasm as a heterodimer [36] and are believed to act together in mRNA degradation and translation, connecting different stages of gene expression [4]. Consistently, Rpb7 was also detectable in polysome fractions (Fig. 2A). However, its presence in these fractions was not dependent upon Not5 in contrast to that of Rpb4, and it was less extensively localized in polysomes than Rpb4 (compare Fig. 2A to Fig. 1C). Furthermore, localization of Rpb7 under the stress conditions, which resulted in a mostly cytoplasmic localization of Rpb4 in wild-type cells, was mostly nuclear and was not affected by Not5 (Fig. 2B). These findings suggest that the presence of Rpb7 in the cytoplasm does not follow the same rules as the presence of Rpb4, neither in the extent of its cytoplasmic localization nor in the Not5-dependence of its polysome association. Moreover, in contrast to Rpb4, Rpb7 does not display a significant two-hybrid interaction with Not5 (Fig. S5).

**Figure 3:**
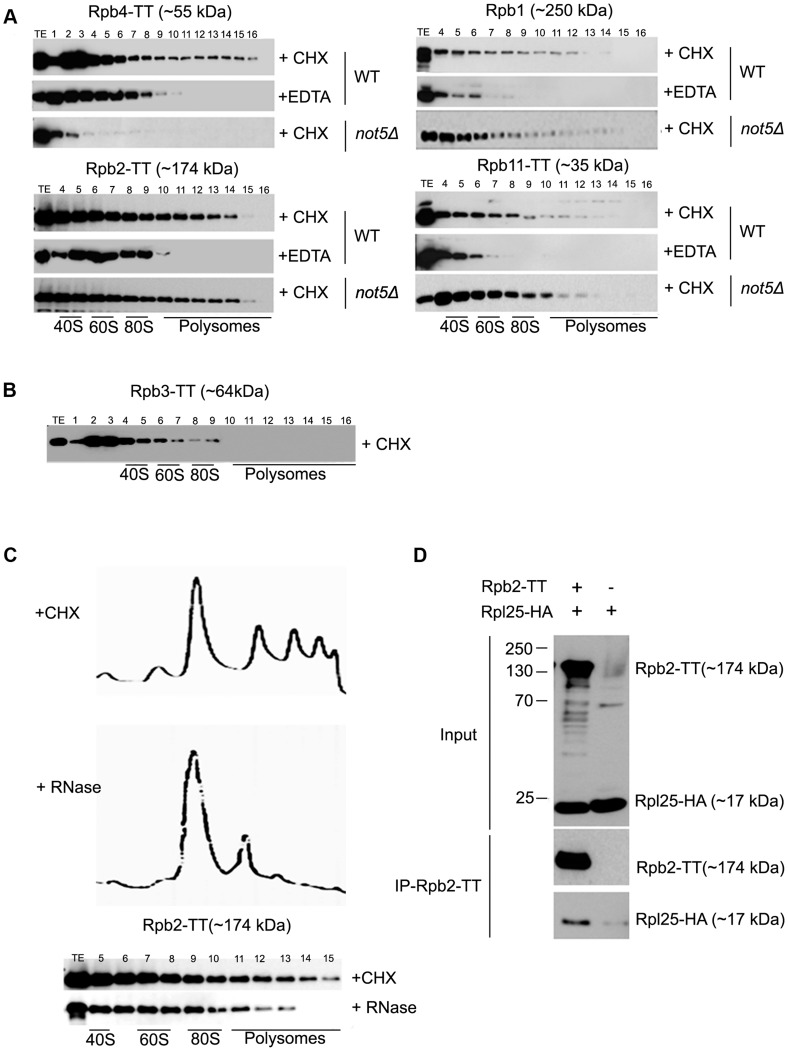
Polymerase subunits are present in polysome fractions and interact with Rpl25. **A** and **B**. Cells expressing Tap-tagged polymerase subunits were analyzed on sucrose gradients as in Fig. 1C. The polysome profiles and protein loading for these experiments are available in Fig. S15. Extracts were treated either with CHX to preserve polysomes or with EDTA to disrupt them or **C**. with RNase to disrupt them. **D**. Rpb2-TT was immunoprecipitated from extracts of cells expressing HA-tagged Rpl25. Cells expressing untagged Rpb2 were used as a control. The immunoprecipitates were incubated with antibodies against HA or CBP to reveal Rpl25 and Rpb2, respectively. The total extract (Input) or immunoprecipitates (IP) were analyzed.

The different behavior of Rpb4 and Rpb7 concerning the extent of their polysome association and its dependence upon Not5 led us to study other RNA Pol II subunits in this respect. The other subunits that we investigated, including the two largest subunits, Rpb1 and Rpb2, as well as the RNA pol II-specific subunit Rpb11, were present in polysome fractions regardless of the presence or absence of Not5 but dependent upon polysome integrity as defined by polysome disruption with EDTA (Fig. 3A). The exception was Rpb3, which was not detectable in polysome fractions (Fig. 3B) as has previously been described [4].

These unexpected findings led us confirm that RNA Pol II subunits interact with ribosomes. We treated cellular extracts with RNase to disrupt polysomes in a manner distinct from EDTA treatment, and confirmed that loss of the heaviest polysomes resulted in the disappearance of Rpb2 from the heaviest fractions (Fig. 3C). We also immunoprecipitated Rpb2 from cells expressing a tagged ribosomal subunit Rpl25 and could co-immunoprecipitate Rpl25 with Rpb2 (Fig. 3D).

**Figure 4:**
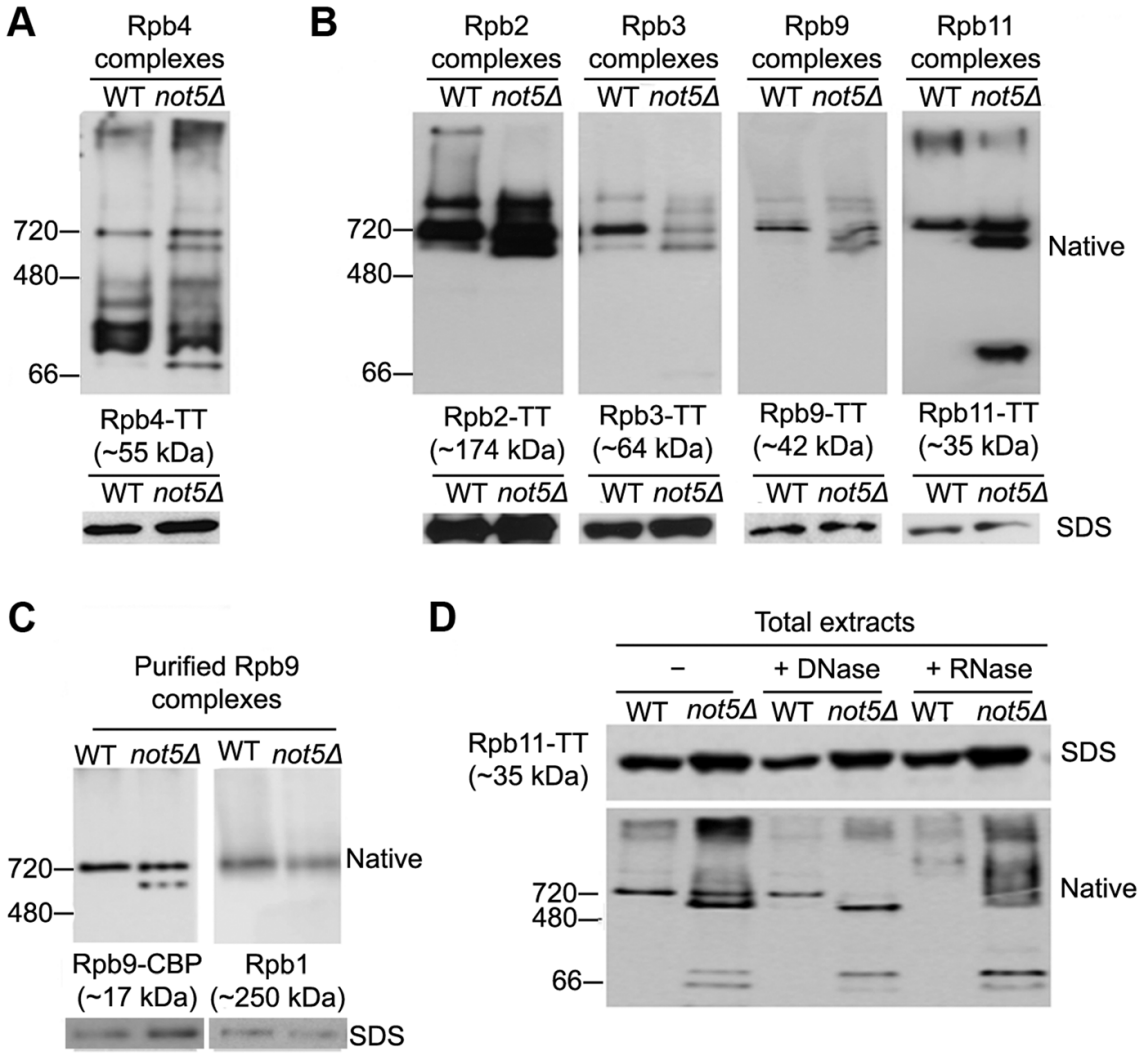
Polymerase sub-complexes lacking Rpb1 accumulate in *not5Δ*. **A** and **B**. Total extracts from cells expressing the indicated Tap-tagged (TT) polymerase subunits were separated on native gels (upper panels) or SDS-PAGE (lower panels) and analyzed by western blotting with anti- CBP antibodies. **C**. Rpb9-TT was purified by single step affinity and the purified proteins were analyzed on native gels (upper panels) or SDS-PAGE (lower panels) and western blotting with anti-CBP antibodies (left panel) or anti-Rpb1 antibodies (right panel). **D**. Total extracts from cells expressing Rpb11-TT were either untreated (-) or treated with DNase or RNase as indicated and separated by Native-PAGE, and analyzed by western blotting with PAP antibodies.

The finding that in addition to Rpb4 and Rpb7, core polymerase subunits are present in polysomes is compatible with reports that RNA Pol II assembly takes place in the cytoplasm, and with the claim that many protein complexes are assembled co-translationally [38]. In addition, since in absence of Not5 Rpb4 fails to localize to polysomes, and does not even accumulate in the cytoplasm, Rpb4 may not assemble optimally with RNA Pol II. To determine whether Rpb4 might dissociate more readily from RNA Pol II in the absence of Not5, we analyzed Rpb4 complexes from total extracts of wild-type or mutant cells on a native gel (Fig. 4A). An Rpb4 complex of the size of core RNA Pol II (around 700 kDa) was detected in both extracts. In addition, many faster migrating forms of Rpb4 were detected, compatible with our knowledge that this subunit readily dissociates from RNA Pol II. However, the extent of these additional smaller complexes was greater in the absence of Not5.

These findings led us to question whether Not5 might be globally affecting RNA Pol II assembly. We looked at complexes of several other RNA Pol II subunits (Rpb2, Rpb3, Rpb9 and Rpb11) from wild-type and mutant cell extracts on native gels. In all cases, a complex of the size of core RNA Pol II was observed with the 4 subunits (Fig. 4B) as with Rpb4 (Fig. 4A). The 4 subunits were detected in at least one additional smaller complex of a similar size in extracts from *not5Δ* (Fig. 4B). These two complexes could readily be purified via any of the 4 subunits as shown for Rpb9 on Fig. 4C. Western blotting revealed that the larger complex contains Rpb1, as expected if it contains core RNA Pol II, but the smaller complex from *not5Δ* lacks Rpb1 (Fig. 4C). This was also observed for the Rpb2, Rpb3 and Rpb11 complexes (Fig. S6). Interestingly, both complexes were sensitive to RNase but not to DNase treatment of the extracts (Fig. 4D) suggesting that they could be newly assembled RNA Pol II or assembly intermediates stabilized with RNA rather than RNA Pol II extracted from chromatin or released after transcription. It is notable that RNase treatment of extracts had an impact on very large forms of the polymerase subunits that without treatment tended to remain at the top of the native gels, or did not even enter the gel (see for instance Rpb11 complexes on Fig. 4B) in both wild-type and mutant extracts. New large heterogeneous forms of the polymerase subunits (shown for Rpb11 on Fig. 4D) were detected in the native gels. It could be that RNase digestion of polysomes released RNA Pol II in forms that could then enter native gels.

Taken together, these results suggest that RNA Pol II assembly is co-translational and that Not5 is important for co-translational assembly of RNA Pol II. This model could be confirmed by pulse labeling wild-type and *not5Δ* cells with ^35^S-methionine and purifying RNA Pol II via Tap-tagged Rpb2 immediately, and after 1 and 2 h chase. We followed the Rpb1 co-purifying with Rpb2. This experiment showed delayed association of labeled Rpb1 with Rpb2 and then subsequently delayed chase of this newly labeled Rpb1 in the Rpb2 purification, in the *not5Δ* strain compared to the wild-type strain (Fig. S7).

### Rpb1 levels are reduced in *not5Δ*

**Figure 5:**
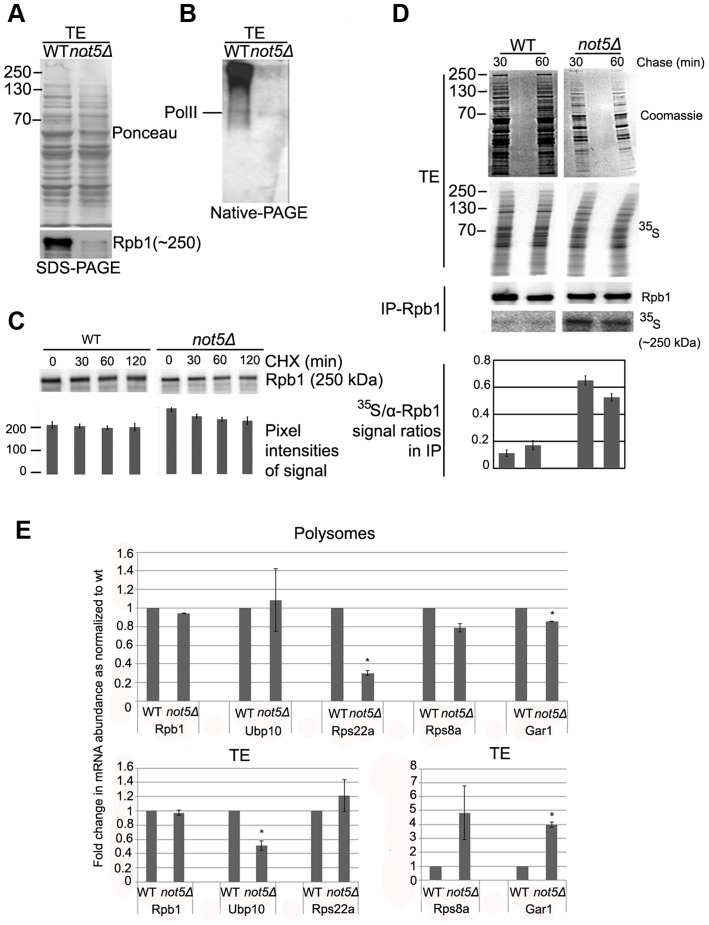
The levels of soluble Rpb1 are decreased in *not5Δ*. **A** and **B**. Total soluble extracts were analyzed by SDS-PAGE (**A**) or Native-PAGE (**B**) and membranes were stained with Ponceau (upper panel in A) or probed with antibodies against Rpb1 (B and lower panel in A). **C**. Cells were grown exponentially to an OD_600_ of 1.0 and then CHX (100 µg ml^−1^) was added. 0.8 OD_600_ units of wild-type cells and 1.6 OD_600_ units of *not5Δ* were collected at the indicated times after protein synthesis arrest. Total proteins prepared by post-alcaline lysis were analyzed by western blotting with antibodies against Rpb1. Quantification of the blots (shown below the blots) revealed no significant difference in the reduction of Rpb1 over time in the 2 strains. **D**. Wild- type and *not5Δ* cells were metabolically labeled with ^35^S for 5 min then chased with cold methionine for 30 and 60 min. Total extracts (TE) were prepared and counted for ^35^S incorporation. The same amount of labeled total protein (20’00 cpm) from both strains (corresponding to 10 µg of protein from wt and 2.5 µg from *not5Δ*) was separated by SDS-PAGE. The gel was stained by Coomassie (TE, Coomassie), dried and then exposed (TE, ^35^S). The same amount of labeled protein from each extract (corresponding to 2 mg of protein from wt and 0.5 mg of protein from *not5Δ*) was also incubated with antibodies against Rpb1, and the immunoprecipitate was analyzed by western blotting (IP-Rpb1, Rpb1). The membranes were also exposed (IP-Rpb1, ^35^S). Quantified ratios of the ^35^S-Rpb1 signal and anti-Rpb1 signal from the western blotting are shown below the blots. **E**. Total extracts from wild-type or *not5Δ* were separated on sucrose gradients as in Fig. 1C, and RNA was extracted from the total extracts (TE, lower panels) or polysome fraction 14 (Fig. S15) (Polysomes, upper panels). The amount of the indicated mRNAs were evaluated by RT followed by qPCR in 1 µg of total and polysomal RNA. Values were normalized to the level of *NIP1* mRNA that showed no change in abundance between the wild-type and *not5Δ* in total extracts or polysome fractions (Fig. S7). All mRNA levels are expressed relative to the level in the total extract of the wild-type expressed as 1. * represents statistically significant differences in mRNA abundance between wild-type and *not5Δ* samples at p<0.05.

The Rpb2, Rpb3, Rpb9 and Rpb11 complexes lacking Rpb1 detected in extracts from cells lacking Not5 (see above) are reminiscent of an RNA Pol II assembly intermediate that has been described [28]. Its accumulation in *not5Δ* cells might indicate that the Rpb1 intermediate, with which it should join, is present in limiting amounts in mutant cells. To address this issue, we compared Rpb1 levels in wild-type and mutant soluble extracts. The level of Rpb1 was lower in extracts from *not5Δ* compared to wild-type, particularly Rpb1 in very large complexes (Fig. 5A and B). Though the difference was not always as dramatic as shown on Fig. 5A and B, it was nevertheless very consistent.

We thus compared the half-life of Rpb1 in wild-type and *not5Δ* cells. No significant reduction of Rpb1 levels after 2 h of protein synthesis arrest was observed suggesting that Rpb1 is not particularly unstable in either wild-type or *not5Δ* cells (Fig. 5C). This finding is in accord with results of a previous study, which measured Rpb1 levels in wild-type cells up to 4 h after protein synthesis arrest [43]. We obtained a similar result after 8 h of protein synthesis arrest (Fig. S8). Hence, increased degradation of Rpb1 is unlikely to explain the low levels of Rpb1 in soluble extracts of *not5Δ*.

*RPB1* mRNA levels are not significantly altered in *not5Δ* cells ([44] and Fig. S4). To determine whether there might nevertheless be a difference in the levels of *de-novo* synthesized Rpb1 between wild-type and *not5Δ* that could explain the reduction of Rpb1 in total extracts of the mutant, we performed a pulse-chase experiment and immunoprecipitated Rpb1 from extracts of labeled cells (Fig. 5D). Surprisingly, for a similar pulse, the amount of soluble proteins labeled was generally higher in *not5Δ* than in wild-type cells. Indeed, the same amount of label was provided by 4 times less total protein from *not5Δ* than from wild-type (compare Coomassie panels of Fig. 5D). Moreover similar amounts of Rpb1 were immunoprecipitated from 4 times less total protein from *not5Δ* than from wild-type (Fig. 5D, IP-Rpb1 upper panel), suggesting that immunoprecipitation of Rpb1 was more efficient from *not5Δ* than from the wild-type. In addition, more of the Rpb1 immunoprecipitated from the mutant was labeled (Fig. 5D, IP-Rpb1 lower panel), indicating that *de-novo* synthesized Rpb1 was more efficiently immunoprecipitated from the mutant, possibly because there was less unlabeled Rpb1 to compete for antibody binding in the mutant. Finally, the amounts of labeled Rpb1 detected in the immunoprecipitate from wild-type or *not5Δ* were not much reduced after 60 min of chase under conditions of protein synthesis arrest. Taken together, these results indicate that newly synthesized Rpb1 is mostly stable and not limiting in *not5Δ* but that it seems to be more accessible to immunoprecipitation in the mutant.

Because of the possible differential competing immunoprecipitation of labeled and unlabeled Rpb1 in the 2 strains in these experiments, we could not definitively define whether translation of *RPB1* mRNA was altered or not in *not5Δ*. We thus checked the relative representation of *RPB1* and other mRNAs in polysomes of *not5Δ* relative to the wild-type. We evaluated the level of *RPB1*, *NIP1* and several other mRNAs in the same amount of RNA prepared from total extracts and polysomes of wild-type and *not5Δ*. We found no significant difference in levels of *RPB1* (Fig. 5E) or *NIP1* (Fig. S9) mRNAs in total extracts or polysomes between wild-type and *not5Δ* cells. In contrast, several mRNAs were significantly reduced in polysomes relative to their representation in total extracts in *not5Δ* compared to the wild-type. For instance levels of *RPS8A* and *GAR1* mRNAs were higher in total extracts but lower in polysomes of *not5Δ* whilst *RPS22A* was expressed at equal levels in both strains, but much less present in polysomes of *not5Δ* (Fig. 5E). In contrast *UBP10* mRNA was under-expressed in *not5Δ* but present at equal levels in polysomes. Taken together, these results indicate that distribution of mRNAs in the translating pool of mRNAs is modified in *not5Δ*.

### Not5 is required for the interaction of the R2TP Hsp90 co-chaperone and Rpb1

If neither the stability nor the *de novo* synthesis of Rpb1 is reduced in *not5Δ* cells, then why are Rpb1 levels in cellular extracts from these cells reduced? The majority of soluble Rpb1 in wild-type extracts is present in heterogeneous complexes, much larger than the major Pol II complex that can be purified via the other Pol II subunits (Fig. 5B). These Rpb1 complexes were severely reduced in *not5Δ* extracts, while the Rpb1-containing complex that was purified via other RNA Pol II subunits (such as Rpb9, see above Fig. 4C) was present in roughly equal amounts in wild-type and mutant cells.

**Figure 6:**
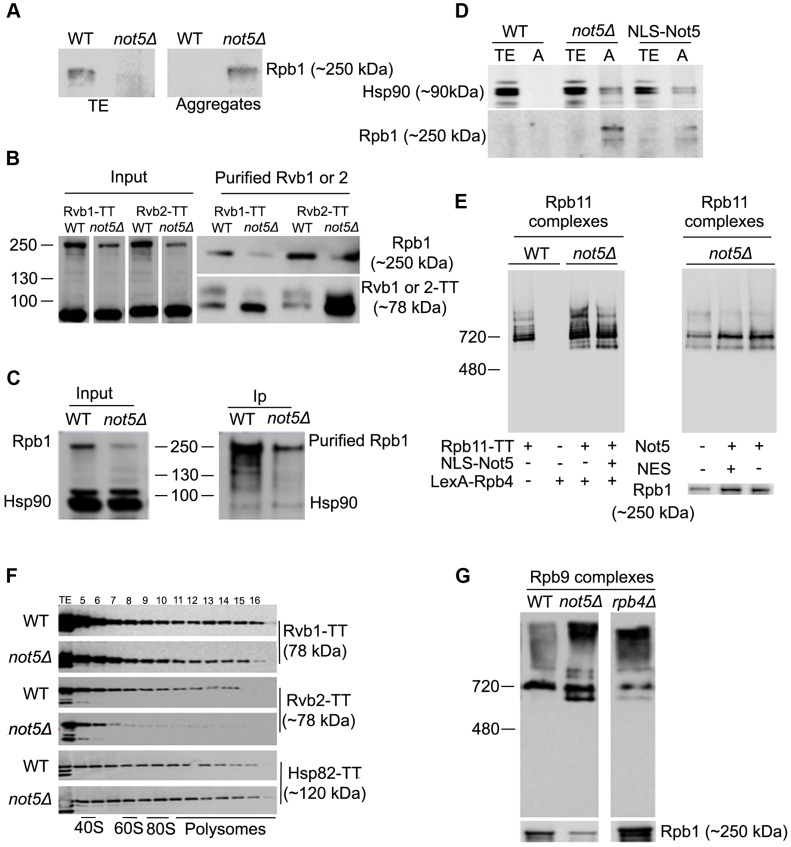
Assembly of Rpb1 with the R2TP Hsp90 co-chaperone is reduced and Rpb1 aggregates in *not5Δ*. **A.** Total extracts and protein aggregates from the indicated strains were analyzed on SDS-PAGE followed by western blotting with antibodies against Rpb1. **B**. Rvb1-TT or Rvb2-TT were purified from wild-type and *not5Δ* cells. Equal amounts of the total extract (Input), and the purified proteins (Purified Rvb1 or 2) were analyzed by western blotting with anti-CBP or anti-Rpb1 antibodies. **C**. Rpb1 was immunoprecipitated from wild-type or *not5Δ* and the level of Rpb1 and Hsp90 proteins in the total extract (Input) immunoprecipitate (Ip) was evaluated by western blotting. **D**. Total extracts (TE) or protein aggregates (A) from wild-type cells (WT) or *not5Δ* cells or from *not5Δ* cells expressing NLS-Not5 as indicated were separated on SDS-PAGE and tested by western blotting for the levels of Hsp90 or Rpb1. **E**. Total extracts from wild-type or *not5Δ* cells grown in galactose expressing Rpb11-TT or not, and expressing or not NLS-Not5 or LexA-Rpb4, as indicated, were separated on native gels and analyzed by western blotting with anti- CBP antibodies (left panel). The same extracts were separated on sucrose gradients and the polysome profiles are shown in Fig. S1A, whereas the distribution of LexA-Rpb4 along the sucrose gradient is shown in Fig. S1B. Total extracts from *not5Δ* cells expressing Rpb11-TT and expressing either Myc-Not5 (Not5) or Myc-Not5-NES (NES) from episomes were separated by Native-PAGE and analyzed by western blotting with anti-CBP antibodies (upper panel) or by SDS-PAGE and analyzed by western blotting with anti-Rpb1 antibodies (lower panel). (**F**). Total extracts from wild-type or *not5Δ* cells expressing Rvb1-TT, Rvb2-TT or Hsp82-TT were analyzed as in Fig. 1C. The polysome profiles for these experiments are available in Fig. S15. **G**. Total extracts from WT, *not5Δ* or *rpb4Δ* cells expressing Rpb9-TT from cells were separated on native gels and analyzed by western blotting with anti-CBP antibodies.

These heterogeneous Rpb1 complexes reduced in *not5Δ* are too large to be mature RNA Pol II, but might include newly synthesized assembly-competent and soluble Rpb1 associated with the Hsp90 chaperone (Hsp82 and Hsc82 in yeast) and the R2TP co-chaperone [28]. If such complexes are reduced in *not5Δ*, one might expect newly produced Rpb1 to fall out of soluble extracts and aggregate. Analysis of total soluble extracts and protein aggregates from wild-type and *not5Δ* showed that this is indeed the case (Fig. 6A). To confirm these observations, we purified the R2TP complex via its subunits Rvb1 and Rvb2, from wild-type and *not5Δ*. The R2TP subunits were expressed at equal levels in both strains, whereas, as expected, the levels of Rpb1 were lower in the mutant (Fig. 6B, left panel). Like Rpb1, the R2TP subunits were purified much more efficiently from *not5Δ* strains (Fig. 6B, right lower panel). Nevertheless Rpb1 co-purified with both Rvb1 and Rvb2 from wild-type cells, but much less from *not5Δ* (Fig. 6B, right upper panel). A similar observation was made when R2TP was purified via the Pih1 subunit (Fig. S10). Whilst interaction of R2TP with Rpb1 appeared reduced in cells lacking Not5, in contrast Rpb1 similarly co-immunoprecipitated Hsp90 (Fig. 6C), and we found that Hsp90, like Rpb1, accumulated in protein aggregates in *not5Δ* (Fig. 6D). Expression of a Not5 derivative that carries a nuclear localization signal and complements the slow growth of *not5Δ*, failed to rescue aggregation of Rpb1 and accumulation of Hsp90 in the aggregates (Fig. 6D), or accumulation of RNA Pol II assembly intermediates (Fig. 6E, left panel), consistent with a role of Not5 in the cytoplasm, at the site of translation. It did however rescue the presence of Rpb4 in polysomes, and it partially rescued polysome levels (Fig. S11A and S11B). In turn, expression of the Not5 derivative that carries a nuclear export signal and that could not rescue accumulation of Rpb4 in the cytoplasm (see Fig. 1H) did rescue levels of soluble Rpb1 and RNA Pol II assembly (Fig. 6E, right panel), and it fully recovered wild-type levels of polysomes (Fig. S11C) but not presence of Rpb4 in polysomes (Fig. S11D).

If R2TP and Hsp90 need to associate with newly produced Rpb1, one might expect that these proteins are also present at the site of translation. We hence tested for the presence of Rvb1, Rvb2 and Hsp82 in polysome fractions of extracts separated on sucrose gradients (Fig. 6F). Indeed, all three proteins were detected in heavy fractions containing polysomes. The presence of these proteins in heavy fractions was clearly dependent in part upon polysome integrity, as determined by disrupting polysomes with EDTA (Fig S1E), supporting the idea that these proteins are associated to some extent with polysomes. Interestingly, in *not5Δ*, while Rvb1 and Hsp82 had sedimentation patterns similar to the wild-type, Rvb2's presence in polysomes was reduced. We also observed that Hsp82 associated significantly with *RPB1* mRNA in wild-type cells, but even more so in the absence of Not5 (Fig. S12). These findings are consistent with a Not5-dependent role in co-translational assembly of R2TP, Hsp90 and Rpb1.

Since Not5 is important for cytoplasmic localization of Rpb4, we wondered whether this could be indirectly the cause for Not5 relevance in appropriate interaction of Rpb1 with R2TP and expression of assembly-competent soluble Rpb1. To address this question, we compared Rpb1 levels in wild-type, *not5Δ* and *rpb4Δ* and how it might affect complexes of other RNA Pol II subunits. The level of Rpb1 was increased in total extracts of *rpb4Δ* rather than decreased as in *not5Δ* and the deletion of Rpb4 had relatively little impact on Rpb9 complexes compared to *not5Δ* (Fig. 6G).

### Rpb1 accumulates in cytoplasmic speckles when Not5 is limiting

**Figure 7:**
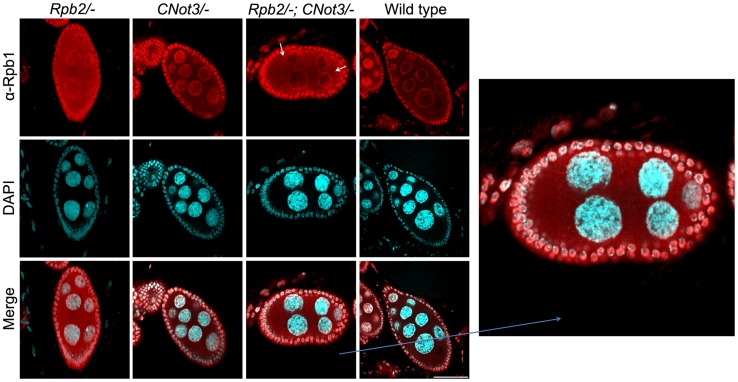
Rpb1 accumulates in cytoplasmic speckles in *RPB2*+/− *CNOT3*+/− trans- heterozygotes. Nurse cells (with large polyploid nuclei surrounded by a layer of follicle cells) of *Drosophila melanogaster* of the indicated genotypes were stained with antibodies against Rpb1, or with DAPI, and the images were merged as indicated. The indicated section was enlarged for better visualization. Scale bar 30 µM.

To test whether aggregation of Rpb1 and the role of Not5 could be demonstrated in living cells in higher eukaryotes, we studied *Drosophila* nurse cells. In these cells active transcription in multiple polythenic nuclei serves the production of the maternal mRNA dowry for the early development of the embryo. We analyzed cells that are heterozygous nulls for *RPB2* and should therefore accumulate cytoplasmic Rpb1 in assembly intermediates, and determined the consequence of making such cells heterozygous nulls for *CNOT3* (the ortholog of yeast *NOT5*). Wild-type cells were used as a control. All cells were stained with antibodies against Rpb1 (Fig. 7). As expected, the absence of one *RPB2* allele led to accumulation of cytoplasmic Rpb1, whilst in wild-type cells Rpb1 was mostly visible in nuclei. In *CNOT3*+/− cells, no major difference could be seen compared to wild-type cells. In trans-heterozygotes, however, cytoplasmic speckles, which correspond probably to Rpb1 aggregates, were clearly visible (Fig. 7 and Fig. S13).

These data indicate that the role of Not5 for the association of newly synthesized Rpb1 with the R2TP co-chaperone to protect newly synthesized Rpb1 from aggregation is likely to be conserved from yeast to flies.

## Discussion

### Not5 links transcription to translation

In this work we show that the Not5 subunit of the Ccr4-Not complex interacts with the Rpb4 subunit of RNA Pol II, and that Not5 is required for the interaction of Rpb4 with the eIF3 translation factor, for Rpb4 presence in polysomes and globally for Rpb4 cytoplasmic localization, because Not5 is required for Rpb4 association with mRNAs. These findings indicate that Not5 contributes importantly to the linkage of transcription to translation by Rpb4.

The mechanism by which Not5 exerts this effect on Rpb4 is unclear. It has been shown that Not5 is recruited to transcribed ORFs, and that the Ccr4-Not complex can interact with RNA Pol II and impact on elongation (for review see [6]). This interaction was first reported not to require Rpb4 [17], but this question is now revisited [45]. We find that localization of Not5 to the nucleus can rescue Rpb4 cytoplasmic accumulation. Therefore we imagine that Not5 contributes either to prevent Rpb4 from dissociating from transcribing RNA Pol II so that it can associate with mRNAs at the completion of transcription or to directly promote Rpb4 association with mRNAs. We demonstrate a very strong interaction of Not5 with Rpb4 in the two-hybrid assay. Similarly, we see strong interaction between Rpb4 and Not3 that is 44% similar to Not5 in its N-terminal domain. Hence, one can assume that Rpb4 interacts with the N-terminal domain of Not5. Consistently, the deletion of this domain of Not5, when combined with the deletion of Not3, leads to a temperature sensitive growth phenotype [46], as does the deletion of Rpb4 [47].

Besides being important for transcription under stress conditions, Rpb4 has also been reported to impact on mRNA export, mRNA degradation and translation [48], [49]. Not5 shares all of these functions, and moreover a recent study reported that single nucleotide changes in *RPB4* or *NOT5* correlate with opposite co-evolution of transcription and mRNA degradation rates [25]. Our current study revealing that Not5 is important for cytoplasmic functions of Rpb4 provides now a good explanation for this functional similarity of the two proteins.

Rpb4 is believed to fulfill its cytoplasmic function as part of the Rpb4-Rpb7 heterodimer. Our results argue against this idea. First, while the interaction of Not5 with Rpb4 is strong, the interaction between Not5 and Rpb7 is weak. Furthermore, while Not5 is important for the cytoplasmic and polysome localization of Rpb4, it is not required for these localizations of Rpb7, which in any event are less prominent than those of Rpb4. It could be that the roles attributed to Rpb7 in the cytoplasm by the analysis of mutant phenotypes are due to its importance to connect Rpb4 with the core RNA Pol II, since this connection is important for the cytoplasmic functions of Rpb4 [4], [35], [36]. The presence of Rpb7 in polysomes, previously argued to indicate a translation function for Rpb7 [4], might instead be related to cytoplasmic co-translational assembly of RNA Pol II. This issue obviously still needs to be clarified.

### Not5 is essential for Rpb1-R2TP interaction and contributes to polymerase assembly

Our study reveals that Not5, besides playing a role in the nucleus for the cytoplasmic localization and cytoplasmic functions of Rpb4, contributes in the cytoplasm to co-translational RNA Pol II assembly. Indeed, we observed that in the absence of Not5, a complex consisting of Rpb2, Rpb3, Rpb9 and Rpb11 but lacking Rpb1 accumulates. This correlates with a critical importance of Not5 for the association of Rpb1 with the R2TP Hsp90 co-chaperone and a tendency of Rpb1 to aggregate in *not5Δ* cells. Interestingly, in the absence of Not5, Hsp90 shows increased association to *RPB1* mRNA and Hsp90 is detected in the *not5Δ* aggregates together with Rpb1. This observation is compatible with a model in which Hsp90 associates with *RPB1* mRNA during Rpb1 production, but in the absence of Not5 has a tendency to remain “stuck” to this mRNA.

The inefficient formation of the soluble assembly-competent Rpb1 intermediate consistently correlates with an accumulation of an Rpb2 intermediate, with which it joins to form RNA Pol II. Though we did not specifically define the composition of this Rpb2 sub-complex, Gpn1/Npa3 that is known to participate in RNA Pol II assembly and to associate with Rpb2, and Iwr1 that binds cytoplasmically assembled RNA Pol II for its nuclear import, were present in similar sub-complexes as Rpb2 in *not5Δ* (Fig. S14).

The importance of Not5 for formation of soluble Rpb1 assembly intermediates does not result from the importance of Not5 for localization of Rpb4 to polysomes. Indeed, the former needs cytoplasmic Not5 that does not rescue presence of Rpb4 in polysomes. Moreover Rpb1 does not aggregate in cells lacking Rpb4, and in fact is present at enhanced levels in extracts from *rpb4Δ*. It is interesting to note that the presence of Rpb4 in polysomes does not entirely rescue polysome levels if Not5 is expressed in the nucleus. Inversely, expression of Not5 in the cytoplasm fully rescues polysome levels despite the absence of cytoplasmic Rpb4.

Our findings argue that Not5 is important for translation at least in two different ways: one via its importance in the nucleus to support Rpb4 presence in the cytoplasm and a second one in the cytoplasm for co-translational events. The exact connection between these roles of Not5 in two separate cellular compartments remains to be determined. The importance of Not5 for translation is further exemplified by a change in specific mRNA translatability.

The importance of Not5 for co-translational RNA Pol II assembly via production of soluble-assembly-competent *de novo* synthesized Rpb1 seems distinct from the reported role of the Bud27 prefoldin, since no reduction of soluble Rpb1 was described in cells lacking Bud27 [34] and it is conserved. Indeed, in *Drosophila* cells, a reduction of the Not5 ortholog, CNot3, results in accumulation of cytoplasmic Rpb1 in speckles.

### Not5 is a major integrator of the different levels of gene expression

Our findings raise the question of how Not5 contributes to the interaction of R2TP with newly synthesized Rpb1, as neither the level of R2TP subunits nor that of newly made Rpb1 is reduced in absence of Not5. We know that Not5 is present at polysomes and interacts with *RPB1* mRNA, and that a component of the Rpb1 assembly complex, in particular Rvb2, is not detectable in polysome fractions in the absence of Not5. Our mass spectrometry analysis of proteins co-purifying with Not5 identified both Rvb1 and Rvb2 (Table S1). Hence Not5 might interact with R2TP subunits and bring them to productively interact with Hsp90 and newly synthesized Rpb1 in ribosomes translating *RPB1* mRNA. This is compatible with the role of the Ccr4-Not complex in co-translational quality control that has been suggested by several recent studies (for review see [50]).

Rvb1 and Rvb2 are not only components of R2TP but also of several important protein complexes (for review see [51]). We observed that both proteins were more accessible for immunoprecipitation in *not5Δ* extracts suggesting that R2TP may be globally more accessible in this strain. It could be that this co-chaperone is globally less well associated with its client proteins, not only with Rpb1. This is compatible with our observation that protein aggregation in *not5Δ* is quite prominent [21] and does not only concern Rpb1, and it is compatible with a reduced presence of Rvb2 in polysomes in the absence of Not5. If true, this would indicate that the cytoplasmic function of Not5 will affect many different protein complexes besides RNA Pol II.

At the same time, Not5 in the nucleus, by being critical for the association of Rpb4 with mRNAs and Rpb4 cytoplasmic functions, will globally affect many different cellular components because Rpb4 has wide-spread roles in translation and mRNA decay. Not5 itself is a component of the same complex as the major yeast deadenylase, and it remains to be defined if the cytoplasmic functions determined for Rpb4 are mediated via its interaction with Not5 in the cytoplasm. In any event, taken together our work identifies Not5 as an essential cellular regulator connecting transcription, mRNA degradation and translation in eukaryotic cells.

## Materials and Methods

### Strains and plasmids

The *Saccharomyces cerevisiae* strains used in this work are listed in Table S2. The plasmid encoding NLS-Not5 (pJG4-5-*NOT5*) has already been described [46]. To generate a plasmid expressing Myc-tagged Not5 we used pGREG516 [52] and inserted the *RPS7A* promoter and *NOT5* coding sequences by the drag and drop technique leading to pMAC763. To fuse a NES (LALKLAGLDI) at the C-terminus of Not5 we digested pMAC763 with BsiWI and XhoI and amplified *NOT5* sequences with a forward primer (TTT GCC TCA CCC AAC GTC AAT C) located prior to the BsiWI site and a reverse primer: (GAG GTC GAC TTA TAT GTC CAA ACC AGC TAA TTT AAG TGC TAA CAG TTT TTC GAA ATC TTC TTC AT) including a SalI site, a stop codon, the NES sequence and the end of the *NOT5* ORF, digested this fragment with BsiWI and SalI and cloned it to the BsiWI and XhoI site of the digested pMAC763. The plasmid obtained was verified by sequencing.

### Polysome fractionation and RNA preparation from polysomes

Ribosomes were fractionated on a 12 ml 7–47% sucrose gradient as in [21]. To analyze comparable amounts of polysomes we applied 2 mg of wild-type and 4 mg of *not5Δ* extracts. The polysomes were disrupted by adding 25 mM EDTA or by 1 µg/ml RNaseA and incubation for 5 min at room temperature. RNasin Plus (Promega) at 0.2 unit/µl was added to stop the nuclease digestion. RNA was isolated from heavy polysome fractions by the Trizol reagent (Invitrogen) following the recommendations of the manufacturer, pellets were washed two times by 75% ethanol to remove sucrose, RNA concentration was measured by nanodrop.

### Two-hybrid experiments

These assays were performed as described [53], [54]. Relevant ORFs were amplified by PCR and cloned into pJG4-5 and pLex202. The relevant combination of plasmids was transformed into MY290.

### Isolation of protein aggregates

Aggregated proteins from total extracts were isolated as previously described [21].

### Metabolic pulse-labeling

One hundred ml of cells grown exponentially to an OD_600_ of 0.8 were resuspended in 50 ml of medium lacking methionine and incubated for 15 min at 30°C with agitation. Cells were pelleted and resuspended in 5 ml of the same medium and then incubated in the presence of 5 µCi/OD_600_ unit of ^35^S methionine for 5 min at 30°C. To stop the labeling reaction, 25 ml of ice cold H_2_O with (for Rpb1 immunoprecipitation) or without (for Rpb2 purification) 100 µg ml^−1^ of CHX was added to the reaction. Both labeling experiments were done in biological duplicates. Cells were pelleted, resuspended in YPD with or without 100 µg ml^−1^ of CHX and placed at 30°C. 10 ml of cells were pelleted and frozen at 30 and 60 min for Rpb1 immunoprecipitation or at 60 and 120 min for Rpb2 purification, for extract preparation. 5 µl of total extracts at 2 mg/ml were TCA precipitated with 300 µl of ice-cold 25% TCA containing 2% of casamino acids for 30 min on ice. The precipitate was collected by vacuum filtering of 250 µl the TCA reaction mix on Whatman GF/A glass fiber filters. Amino acids were removed by rinsing the filter 3 times with 1 ml of ice-cold 5% TCA. For determination of ^35^S incorporation into translation products the filter was put into scintillation fluid (NOCS 104; Amersham) and counted in a Wallac 1409 liquid scintillation counter. The same amount of labeled protein from each extract in a volume of 800 µl was incubated with 0.5 µg anti-CTD antibody and magnetic Protein G beads (Invitrogen) for Rpb1 immunoprecipitation or directly with IgG beads for Rpb2 purification, that were pretreated with 200 µl of 5 mg/ml *not5Δ* total protein extracts in IP buffer (40 mM HEPES-KOH pH 8.0, 100 mM KCl, 150 mM potassium acetate, 1 mM EDTA, 10% glycerol and protease inhibitors) to saturate unspecific binding. For Rpb1 immunoprecipitation, after 3 washes beads were boiled with 50 µl of SB, and for Rpb2 purification the beads were washed additionally 3 times with TEV cleavage buffer and exposed to TEV cleavage as described below. Both preparations were analyzed by western blotting and by Coomassie staining. Coomassie stained gels were dried and revealed by Phosphorimager (Typhoon Phosphorimager 8600).

### RNA-Immunoprecipitation (RIP) and qPCR

One hundred milliliter of cells collected at OD_600_ 0.8–1.0 was treated with CHX (100 µg ml^−1^) for 10 min at 4°C and cells were harvested. Single-step affinity purification was done in the presence of CHX (100 µg ml^−1^) and 80 units/ml of RNase inhibitor (RNasine, Fermentas). RNase inhibitor and CHX were applied in similar concentrations for all the subsequent steps of the RIP. TEV cleavage was done in IP buffer (described above) for 1 h at 30°C. One fourth of the TEV eluate and 25 µg of total protein were subjected to western blotting to verify the affinity purification. 0.5 mg of total extracts and the rest of the TEV eluates were treated with phenol-chloroform for nucleic acid extraction. The nucleic acids were precipitated at -20°C with ethanol upon addition of sodium-acetate (100 mM) and 3 µl of linear acrylamide (Fermentas). Pellets were resuspended in H_2_O and were DNaseI treated (RQ1 RNase-free DNase, Promega). For RIPs, 500 ng of the TEV eluates and 500 ng of the RNA from the total extracts were reverse transcribed, for polysomal and total RNA comparisons 1 µg of RNA were reverse transcribed, with M-MLV RT (Promega) using oligo d(T) primers according to manufacturer's instructions. In performing the DNase and reverse transcription experiments we followed the manufacturer's instructions. qPCR primers were constructed to amplify approximately 200 bp long fragments close to the polyA tail of the mRNA. As a positive control we used Rpl17-TT to immunoprecipitate all translated mRNAs. Negative control was a wild-type strain without any protein tagged. We conducted qPCR on the reverse transcribed samples. For each 20 µl reaction, 9 µl first strand cDNA solution, 10 µl ABsolute qPCR SYBR Green Mix (ABgene), 0.5 µl forward primer (10 µM) and 0.5 µl reverse primer (10 µM) were mixed together. PCR parameters were as follows; 95°C, 10 min for heat activation of DNA polymerase mix, followed by 94°C, 15 s (denaturation); 60°C, 1 min (annealing and synthesis) for 38 cycles. Relative enrichment ratios and relative mRNA abundances were determined by the Pfaffl method [55], and normalized to the total RNA input in the case of the RIPs, and to wild-type RNA levels in the case of the polysome-total RNA comparisons in which *NIP1* mRNA was used as a loading control. The primers used are: Rpb1 5′: GTC ACC AAG TTA CAG CCC AAC G; Rpb1 3′: AGA TCC TGG GCT GTA GCC TG; Nip1 5′: AGC TGA TGA GCG TGC TAG AC; Nip1 3′: AGG AAC GAC GAA TGG ATT TTG GAG.

### Immunofluorescence

Cells were grown to a log phase (OD_600_ of 0.5), fixed by adding 1 ml of 37% formaldehyde and incubating for 2 h at RT, then pelleted, washed with PBS (10 mM sodium phosphate buffer pH 7.4, 137 mM NaCl, 2.7 mM KCl) and resuspended in 0.5 ml of spheroplasting buffer (20 mM potassium phosphate pH 7.4, 1.2 M sorbitol). 0.2 ml of cell suspension was treated with 3.2 µl of 1.42 M β-mercaptoethanol and 5 µl of 5 mg/ml zymolyase 100T for 60 min at 30°C. Cells were washed once with 1 ml then resuspended in 100 µl of PBS +0.05% Tween 20. 20 µl of spheroplasts were placed on polylysine coated microscope slides and dried. Slides were washed three times with PBS and cells were blocked in 20 µl of PBS containing 1 mg/ml BSA for 30 min. 20 µl of primary antibody diluted 1∶200 in PBS with BSA was placed on the cells and incubated for 1 h at RT. Cells were washed three times with PBS and incubated for 1 h at RT with secondary antibody diluted 1∶1000 in PBS with BSA. After 3 washes with PBS, cells were treated with 20 µl of 1 µg/ml DAPI in PBS and washed again 3 times with PBS. Glass slides were mounted in 90% glycerol containing PBS and analyzed with fluorescent microscopy using an Axio Vert 200 device supplied with cooled CCD camera or with a Nikon Ti-E motorized inverted microscope system equipped with an Orca-Flash 4.0 Digital CMOS camera.

The *Drosophila* P-element insertion lines used for immunofluorescence were obtained from the Bloomington stock center (stock numbers: 15271 for *CNOT3* and 34754 for *RPB2*). The trans-heterozygous flies were generated by crossing the two different P-element insertion lines. As a wild-type control we used the w^1117^ strain. For microscopy *Drosophila* ovaries were fixed in 4% paraformaldehyde. To block nonspecific staining embryos were incubated in 1% BSA (Sigma) in PBST (0.1% (v/v) Tween 20 in PBS) for 120 min at 4°C. Ovaries were incubated with the primary antibodies and 1% BSA in PBST. After several rinses in PBST, ovaries were incubated in secondary antibodies for 3 h at room temperature. To detect DNA, ovaries were stained with DAPI following incubation with the secondary antibody. Following several rinses in PBST ovaries were mounted in Aqua Poly Mount (Polysciences Inc). Optical sections were generated with an Olympus FV1000 confocal microscope.

### Co-immunoprecipitations and affinity purifications

For small scale tandem affinity purifications of RNA Pol II or R2TP 100 OD_600_ units of cells were broken with 0.5 ml of glass beads in 0.6 ml of IP buffer during 25 min at 4°C. Beads were washed with 0.5 ml of IP buffer. After clarification, 0.8 ml of the supernatants containing 4 mg of total protein treated when indicated with 1 µg/ml RNaseA for 5 min at room temperature were incubated with 40 µl of IgG sepharose beads (GE Healthcare). The beads were washed three times with 0.2 ml of IP buffer and then 3 times with 0.2 ml TEV buffer (10 mM Tris-HCl, pH 8.0, 150 mM NaCl, 0.1% Tween 20, 0.5 mM EDTA). Beads were incubated for 1 h at 30°C in 40 µl of TEV buffer containing 1 µM DTT and 1 unit of TEV protease (Invitrogen). Beads were sedimented and the supernatant was applied for native 3–12% Bis-Tris gel (Invitrogen) analysis or boiled with SDS sample buffer (SB) and separated by SDS-PAGE (4–12%) followed in both cases by western blotting.

### Native gel analysis

RNA Pol II was single-step purified from 4 mg of total protein obtained from 100 OD_600_ units of cells. Eluates were concentrated to 25 µl and analyzed by Native PAGE 3–12% Bis-Tris gels (Invitrogen). From total extracts 25 µg proteins were analyzed by Native PAGE. When indicated total extracts were treated either by 1 µg/ml RNaseA for 5 min at RT, or by 20 units/ml of DNase I (New England BioLabs Inc.) for 10 min at 37°C. After Native PAGE samples were analyzed by western blotting.

### Antibodies

Primary antibodies used for western blotting were anti CBP (Anti-Calmodulin Binding Protein; DAM1411288; Millipore) used at 1∶5000, anti HA (Anti influenza hemagglutinin; H3663; Sigma) used at 1∶5000, anti CTD which recognizes Rpb1 and will be referred to in the text as anti Rpb1 (ab5408; Abcam) used at 1∶500, or finally anti PAP (Peroxidase-Anti-Peroxidase; P1291; Sigma) used at 1∶10000 and rabbit polyclonal anti Ccr4 which was generated in our laboratory and used at 1∶5000. The secondary antibodies were anti-Mouse-HRP (IgG-Peroxidase conjugate; A9044; Sigma) used at 1∶10000 or anti-Rabbit-HRP (IgG-Peroxidase conjugate; A8275; Sigma) used at 1∶10000. For detection of Rpb1, ovaries were incubated with 7G5 mouse monoclonal antibody used at 1∶1000 (a kind gift of Dr. László Tora, Strasbourg).

## Supporting Information

Figure S1 **A**. Serial dilutions of exponentially growing reporter cells expressing LexA-Rpb4 as a bait, and the indicated proteins fused to B42 as preys, were spotted on medium selective for the plasmids and indicative of an interaction between bait and prey. **B**. Upper panels: Rpb4-TT was immunoprecipitated from cells expressing Rpb4-TT and the presence of Rpb4 and Ccr4 was evaluated by western blotting with antibodies against CBP and Ccr4 respectively. A strain lacking any Tap-tagged protein was used as a control (No-TT). Immunoprecipitation with RNase-treated samples are also shown. Lower panels: Not4-TT was immunoprecipitated from cells expressing HA-tagged Rpb4 and Not4-TT. The presence of Not4 and Rpb4 in the total extract (TE), flow through (FT) and immunoprecipitate (IP-TT) was analyzed by western blotting with antibodies against CBP and HA respectively. A strain expressing HA-tagged Rpb4 but no Tap-tagged protein was used as a control (No-TT). Immunoprecipitation with RNase treated samples are also shown. **C**. Serial dilutions of exponentially growing cells from the indicated strains were spotted on plates and left to grow for several days at 30°C. **D**. Serial dilutions of exponentially growing reporter cells expressing LexA-Nip1 as a bait, and the indicated proteins fused to B42 as preys, were spotted either on medium selective for the plasmids and indicative of an interaction between bait and prey. **E**. Fractions from 7–47% sucrose gradients of extracts treated or not with EDTA as indicated from wild-type or *not5Δ* expressing Tap-tagged Not1, Rvb1 and Hsp82 were precipitated with TCA and analyzed by western blotting with PAP antibodies. The positions of 40S, 60S, 80S and polysomes are indicated under the blots. The numbers of the gradient fractions tested or the total extract (TE) are indicated at the top. The strains used that are not included in our strain list are MY4856 (Isogenic to BY4741 except *MAT*α *not1::NOT1-Taptag-URA3)*, MY5277 (*MAT*a *leu2Δ20 ura3Δ met15Δ his3Δ 1 ccr4::HIS3 lys2Δ0*), MY5676 (*MAT*a *leu2Δ20 ura3Δ met15Δ his3Δ1 not5::NATMX4 not1::NOT1-TapTag-URA3*), MY8768 (*MAT*a *his3Δ1 leu2Δ0 lys2 Δ0 ura3Δ0 rpb4::KanMX4*), MY8984 (*MAT*a *rpb4::KanMX4 caf40::HIS3MX4 lys2*), MY9080 (*his3Δ1 leu2Δ0 lys2Δ0 ura3Δ0 ccr4::HIS3MX4 rpb4::KanMX4*), MY10913 (*MAT*a *his3Δ leu2Δ lys2Δ0 ura3Δ caf40::NATMX4*), MY8853 (*MAT*α *leu2Δ20 ura3Δ met15Δ lys2Δ0 his3Δ1 not4::NOT4-TAPTAG-URA3 rpb4::RPB4-HA3-KanMX4*) and MY9167 (*MAT*α *rpb4::RPB4-HA3-KanMX4 his3*). Plasmids expressing B42-Rpb4, B42-Rpb7 and LexA-Nip1 were created by PCR amplification followed by restriction enzyme digestion and ligation into predigested empty vectors.
(PDF)

Figure S2 *not5Δ* cells expressing Rpb4-TT and the indicated Not5 derivatives, were grown exponentially and stained with anti-Myc antibodies (middle panels), or DAPI (right panels) or the pictures were merged (left panels).
(PDF)

Figure S3 Rpb4 levels are the same in wt and *not5Δ* cells and Rpb4 immunoprecipitated to similar extent from both strains. TE: total extracts, Purif: Tap tag purification followed by TEV cleavage.
(PDF)

Figure S4 Rpb4 and Not5 are associated with *RPB1* mRNA. The indicated Tap-tagged proteins were immunoprecipitated from total extracts of wild-type or *not5Δ* cells and RNA was purified from total extracts (TE) and the immunoprecipitates (RIP). The levels of *RPB1* or *NIP1* mRNA in 0.5 µg of TE and RIP RNA were measured by real-time PCR and expressed relative to the amount of these mRNAs identified in the TE of the wild-type (expressed as 1). * represents statistically significant enrichment of the RNA in the RIP relative to the TE at p<0.05. The strains used absent in our main strain list are MY5321 (Isogenic to BY4741 except *not5::NOT5-TapTag-KanMX4*) and MY9632 (*MAT*a *ade2 arg4 leu2,3112 trp1-289 ura3-52 rpl17b::RPL17B*-*Taptag*-*URA3*; from Euroscarf).
(PDF)

Figure S5 Rpb7 interacts with Not3 and Not5 to a lesser extent than Rpb4. 10-fold serial dilutions of exponentially growing reporter cells expressing LexA-Rpb4 or LexA-Rpb7 as a bait, and the indicated proteins fused to B42 as preys, were spotted on medium selective for the plasmids and indicative of an interaction between bait and prey. Note that Rpb7 interaction with Not3 or Not5 is weaker than that of Rpb4 if compared to known interaction with Nip1.
(PDF)

Figure S6 A lower molecular weight RNA Pol II complex lacking Rpb1 can be purified via several different RNA Pol II subunits. The indicated Tap-tagged RNA Pol II subunits were purified by a single step affinity purification and the purified proteins were analyzed on native gels and western blotting with anti-CBP antibodies (left panel) or anti-Rpb1 antibodies (right panel). The complex of a size compatible with mature RNA Pol II and a subcomplex enriched in *not5Δ* (*) are indicated.
(PDF)

Figure S7 Delayed association of newly produced Rpb1 with Rpb2 in *not5Δ*. Exponentially growing wild-type and *not5Δ* cells expressing Rpb2-TT were pulse-labeled for 5 min with 35S-methionine (Met). We adjusted the input extracts to obtain similar levels of purified labeled proteins from both strains. We collected samples for Rpb2-TT right after the 5 min pulse, or 60 or 120 min after the chase. Rpb2 was purified by immunoaffinity followed by TEV cleavage. Eluates from the different time points were analyzed by western blotting with antibodies to Rpb1 (α-Rpb1) or with Phosphoimager (^35^S-Rpb1) to visualize the radioactive signal. Quantified ratios of the signal of ^35^S-Rpb1 relative to the signal of α-Rpb1 from the western blot are shown below the blots.
(PDF)

Figure S8 Rpb1 levels are stable even after 8 hours of protein synthesis arrest both in wt and *not5Δ* cells. Cells were grown exponentially to an OD_600_ of 1.0 and then CHX (100 µg ml^−1^) was added. 0.8 OD_600_ units of wild-type cells and 1.6 OD_600_ units of *not5Δ* were collected at the indicated times after protein synthesis arrest. Total proteins prepared by post-alcaline lysis were analyzed by western blotting with antibodies against Rpb1.
(PDF)

Figure S9 Similar *NIP1* mRNA levels in wild-type and *not5Δ*. Total extracts from wild-type or *not5Δ* were separated on sucrose gradients as in Fig. 1C, and RNA was extracted from the total extracts (TE, left panel) or polysome fraction 14 (Fig. S15) (Polysomes, right panel). The amount of *NIP1* mRNA was evaluated by RT followed by qPCR in 1 µg of total and polysomal RNA. The experiment was repeated 4 times and revealed no statistical significant difference of *NIP1* mRNA levels in TE or polysomes of the wild-type compared to *not5Δ*.
(PDF)

Figure S10 Less Rpb1 co-purifies with Pih1 from *not5Δ* than from wild type cells. The same experiment as in Figure 6B was performed with cells expressing Tap-tagged Pih1.
(PDF)

Figure S11 **A**. The total extracts from the 4 strains presented in Fig. 6E, left panel were separated on sucrose gradients and the polysome profiles are presented. **B**. Proteins in the different fractions of the wild-type and *not5Δ* +NLS-Not5 strains expressing LexA-Rpb4 were TCA precipitated and analyzed by western blotting to reveal the presence of LexA-Rpb4. **C**. The total extracts from the 3 strains presented in Fig. 6E, right panel were separated on sucrose gradients and the polysome profiles are presented. **D**. Proteins in the different fractions of the *not5Δ* + Myc-Not5 or *not5Δ* + Myc-Not5-NES strains expressing Rpb4-TT were TCA precipitated and analyzed by western blotting to reveal the presence of Rpb4-TT.
(PDF)

Figure S12 Hsp82 association with *RPB1* mRNA is increased in cells lacking Not5. Tap-tagged Hsp82 was immunoprecipitated from total extracts of wild-type or *not5Δ* cells and RNA was purified from total extracts (TE) and the immunoprecipitates (RIP). The levels of *RPB1* or *NIP1* mRNA in 0.5 µg of TE and RIP RNA were measured by real-time PCR and expressed relative to the amount of these mRNAs identified in the TE of the wild-type (expressed as 1). * represents statistically significant enrichment of the RNA in the RIP relative to the TE at p<0.05.
(PDF)

Figure S13 Egg chambers of different developmental stages ranging from stage 4 to stage 10 of *Drosophila melanogaster* of the indicated genotypes were stained with antibodies against Rpb1 or with DAPI, and the images were merged, as indicated.
(PDF)

Figure S14 Total extracts from wild-type or *not5Δ* cells expressing the indicated Tap-tagged (TT) proteins were separated on native gels and analyzed by western blotting with anti-CBP antibodies. The strains 10269 (*MAT*a *ade2 arg4 leu2,3112 trp1-289 ura3-52 iwr1::IWR1-Taptag-URA3*) and 10427 (*MAT*a *iwr1::IWR1-Taptag-URA3 not5::LEU2*) and MY10266 (*MAT*a *ade2 arg4 leu2,3112 trp1-289 ura3-52 npa3::NPA3-Taptag-URA3*) and MY10436 (*MAT*a *npa3::NPA3-Taptag-URA3 not5::LEU2*) were used.
(PDF)

Figure S15 The different sucrose gradient fractionations shown in the manuscript are presented. On the top is presented a western-blot showing the distribution of Rps3, a ribosomal protein of the small ribosomal subunit, along a typical sucrose gradient of wt and *not5Δ* in this manuscript.
(PDF)

Figure S16 Prt1-HA is not immunoprecipitated non-specifically from WT or *not5Δ* cells. WT or *not5Δ* cells expressing Not1-TT or not expressing any Tap-tagged protein (No TT), and expressing Prt1-HA were incubated with IgG sepharose beads, washed and specifically bound proteins were eluted and analyzed by SDS-PAGE followed by western blotting with antibodies against CBP for the TT proteins or HA for Prt1-HA.
(PDF)

Table S1 Rvb1 and Rvb2 are identified with various peptides in Not5 purification with LC/MS/MS. Tap-tagged Not5 was purified and the purified proteins were loaded on a native gel that was stained with Commassie. The entire lane was cut in slices and analyzed by LC/MS/MS. Table shows the polypeptides that identified Rvb1 and Rvb2 as co-purifying proteins with Not5.
(DOCX)

Table S2 Yeast strains used in this work.
(DOCX)

## References

[1] Hsin JP, Manley JL (2012) The RNA polymerase II CTD coordinates transcription and RNA processing. Genes & development 26: 2119–2137. 23028141 10.1101/gad.200303.112 PMC3465734

[2] Aguilera A (2005) Cotranscriptional mRNP assembly: from the DNA to the nuclear pore. Current opinion in cell biology 17: 242–250. 15901492 10.1016/j.ceb.2005.03.001

[3] de Almeida SF, Carmo-Fonseca M (2008) The CTD role in cotranscriptional RNA processing and surveillance. FEBS letters 582: 1971–1976. 18435923 10.1016/j.febslet.2008.04.019

[4] Harel-Sharvit L, Eldad N, Haimovich G, Barkai O, Duek L, et al (2010) RNA polymerase II subunits link transcription and mRNA decay to translation. Cell 143: 552–563. 21074047 10.1016/j.cell.2010.10.033

[5] Goler-Baron V, Selitrennik M, Barkai O, Haimovich G, Lotan R, et al (2008) Transcription in the nucleus and mRNA decay in the cytoplasm are coupled processes. Genes & development 22: 2022–2027. 18676807 10.1101/gad.473608 PMC2492753

[6] Collart MA, Panasenko OO (2012) The Ccr4–not complex. Gene 492: 42–53. 22027279 10.1016/j.gene.2011.09.033

[7] Collart MA (2003) Global control of gene expression in yeast by the Ccr4-Not complex. Gene 313: 1–16. 12957374 10.1016/s0378-1119(03)00672-3

[8] Albert TK, Lemaire M, van Berkum NL, Gentz R, Collart MA, et al (2000) Isolation and characterization of human orthologs of yeast CCR4-NOT complex subunits. Nucleic acids research 28: 809–817. 10637334 10.1093/nar/28.3.809 PMC102560

[9] Temme C, Zhang L, Kremmer E, Ihling C, Chartier A, et al (2010) Subunits of the Drosophila CCR4-NOT complex and their roles in mRNA deadenylation. RNA 16: 1356–1370. 20504953 10.1261/rna.2145110 PMC2885685

[10] Lau NC, Kolkman A, van Schaik FM, Mulder KW, Pijnappel WW, et al (2009) Human Ccr4-Not complexes contain variable deadenylase subunits. The Biochemical journal 422: 443–453. 19558367 10.1042/BJ20090500

[11] Bawankar P, Loh B, Wohlbold L, Schmidt S, Izaurralde E (2013) NOT10 and C2orf29/NOT11 form a conserved module of the CCR4-NOT complex that docks onto the NOT1 N-terminal domain. RNA biology 10 10.4161/rna.23018 PMC3594282 23303381

[12] Mauxion F, Preve B, Seraphin B (2012) C2ORF29/CNOT11 and CNOT10 form a new module of the CCR4-NOT complex. RNA biology 10 10.4161/rna.23065 PMC3594285 23232451

[13] Farber V, Erben E, Sharma S, Stoecklin G, Clayton C (2013) Trypanosome CNOT10 is essential for the integrity of the NOT deadenylase complex and for degradation of many mRNAs. Nucleic acids research 41: 1211–1222. 23221646 10.1093/nar/gks1133 PMC3553956

[14] Deluen C, James N, Maillet L, Molinete M, Theiler G, et al (2002) The Ccr4-not complex and yTAF1 (yTaf(II)130p/yTaf(II)145p) show physical and functional interactions. Molecular and cellular biology 22: 6735–6749. 12215531 10.1128/MCB.22.19.6735-6749.2002 PMC134042

[15] Swanson MJ, Qiu H, Sumibcay L, Krueger A, Kim SJ, et al (2003) A multiplicity of coactivators is required by Gcn4p at individual promoters in vivo. Molecular and cellular biology 23: 2800–2820. 12665580 10.1128/MCB.23.8.2800-2820.2003 PMC152555

[16] Venters BJ, Wachi S, Mavrich TN, Andersen BE, Jena P, et al (2011) A comprehensive genomic binding map of gene and chromatin regulatory proteins in Saccharomyces. Molecular cell 41: 480–492. 21329885 10.1016/j.molcel.2011.01.015 PMC3057419

[17] Kruk JA, Dutta A, Fu J, Gilmour DS, Reese JC (2011) The multifunctional Ccr4-Not complex directly promotes transcription elongation. Genes & development 25: 581–593. 21406554 10.1101/gad.2020911 PMC3059832

[18] Lenssen E, James N, Pedruzzi I, Dubouloz F, Cameroni E, et al (2005) The Ccr4-Not complex independently controls both Msn2-dependent transcriptional activation—via a newly identified Glc7/Bud14 type I protein phosphatase module—and TFIID promoter distribution. Molecular and cellular biology 25: 488–498. 15601868 10.1128/MCB.25.1.488-498.2005 PMC538800

[19] Tucker M, Valencia-Sanchez MA, Staples RR, Chen J, Denis CL, et al (2001) The transcription factor associated Ccr4 and Caf1 proteins are components of the major cytoplasmic mRNA deadenylase in Saccharomyces cerevisiae. Cell 104: 377–386. 11239395 10.1016/s0092-8674(01)00225-2

[20] Wahle E, Winkler GS (2013) RNA decay machines: Deadenylation by the Ccr4-Not and Pan2-Pan3 complexes. Biochimica et biophysica acta 1829: 561–570. 23337855 10.1016/j.bbagrm.2013.01.003

[21] Panasenko OO, Collart MA (2012) Presence of Not5 and ubiquitinated Rps7A in polysome fractions depends upon the Not4 E3 ligase. Molecular microbiology 83: 640–653. 22243599 10.1111/j.1365-2958.2011.07957.x

[22] Dimitrova LN, Kuroha K, Tatematsu T, Inada T (2009) Nascent peptide-dependent translation arrest leads to Not4p-mediated protein degradation by the proteasome. The Journal of biological chemistry 284: 10343–10352. 19204001 10.1074/jbc.M808840200 PMC2667721

[23] Halter D, Collart MA, Panasenko OO (2014) The Not4 E3 ligase and CCR4 deadenylase play distinct roles in protein quality control. PloS one 9: e86218. 24465968 10.1371/journal.pone.0086218 PMC3895043

[24] Panasenko OO, Collart MA (2011) Not4 E3 ligase contributes to proteasome assembly and functional integrity in part through Ecm29. Molecular and cellular biology 31: 1610–1623. 21321079 10.1128/MCB.01210-10 PMC3126335

[25] Dori-Bachash M, Shema E, Tirosh I (2011) Coupled evolution of transcription and mRNA degradation. PLoS biology 9: e1001106. 21811398 10.1371/journal.pbio.1001106 PMC3139634

[26] Cramer P (2002) Multisubunit RNA polymerases. Current opinion in structural biology 12: 89–97. 11839495 10.1016/s0959-440x(02)00294-4

[27] Wild T, Cramer P (2012) Biogenesis of multisubunit RNA polymerases. Trends in biochemical sciences 37: 99–105. 22260999 10.1016/j.tibs.2011.12.001

[28] Boulon S, Pradet-Balade B, Verheggen C, Molle D, Boireau S, et al (2010) HSP90 and its R2TP/Prefoldin-like cochaperone are involved in the cytoplasmic assembly of RNA polymerase II. Molecular cell 39: 912–924. 20864038 10.1016/j.molcel.2010.08.023 PMC4333224

[29] Boulon S, Bertrand E, Pradet-Balade B (2012) HSP90 and the R2TP co-chaperone complex: building multi-protein machineries essential for cell growth and gene expression. RNA biology 9: 148–154. 22418846 10.4161/rna.18494

[30] Kakihara Y, Houry WA (2012) The R2TP complex: discovery and functions. Biochimica et biophysica acta 1823: 101–107. 21925213 10.1016/j.bbamcr.2011.08.016

[31] Czeko E, Seizl M, Augsberger C, Mielke T, Cramer P (2011) Iwr1 directs RNA polymerase II nuclear import. Molecular cell 42: 261–266. 21504834 10.1016/j.molcel.2011.02.033

[32] Staresincic L, Walker J, Dirac-Svejstrup AB, Mitter R, Svejstrup JQ (2011) GTP-dependent binding and nuclear transport of RNA polymerase II by Npa3 protein. The Journal of biological chemistry 286: 35553–35561. 21844196 10.1074/jbc.M111.286161 PMC3195585

[33] Forget D, Lacombe AA, Cloutier P, Al-Khoury R, Bouchard A, et al (2010) The protein interaction network of the human transcription machinery reveals a role for the conserved GTPase RPAP4/GPN1 and microtubule assembly in nuclear import and biogenesis of RNA polymerase II. Molecular & cellular proteomics: MCP 9: 2827–2839. 20855544 10.1074/mcp.M110.003616 PMC3002788

[34] Miron-Garcia MC, Garrido-Godino AI, Garcia-Molinero V, Hernandez-Torres F, Rodriguez-Navarro S, et al (2013) The prefoldin bud27 mediates the assembly of the eukaryotic RNA polymerases in an rpb5-dependent manner. PLoS genetics 9: e1003297. 23459708 10.1371/journal.pgen.1003297 PMC3573130

[35] Lotan R, Bar-On VG, Harel-Sharvit L, Duek L, Melamed D, et al (2005) The RNA polymerase II subunit Rpb4p mediates decay of a specific class of mRNAs. Genes & development 19: 3004–3016. 16357218 10.1101/gad.353205 PMC1315404

[36] Selitrennik M, Duek L, Lotan R, Choder M (2006) Nucleocytoplasmic shuttling of the Rpb4p and Rpb7p subunits of Saccharomyces cerevisiae RNA polymerase II by two pathways. Eukaryotic cell 5: 2092–2103. 17056745 10.1128/EC.00288-06 PMC1694818

[37] Dahan N, Choder M (2013) The eukaryotic transcriptional machinery regulates mRNA translation and decay in the cytoplasm. Biochimica et biophysica acta 1829: 169–173. 22982191 10.1016/j.bbagrm.2012.08.004

[38] Duncan CD, Mata J (2011) Widespread cotranslational formation of protein complexes. PLoS genetics 7: e1002398. 22144913 10.1371/journal.pgen.1002398 PMC3228823

[39] Collart M, Panasenko O, Nikolaev S (2012) The Not3/5 subunit of the Ccr4-Not complex: A central regulator of gene expression that integrates signals between the cytoplasm and the nucleus in eukaryotic cells. Cellular signalling 10.1016/j.cellsig.2012.12.018 23280189

[40] Maillet L, Tu C, Hong YK, Shuster EO, Collart MA (2000) The essential function of Not1 lies within the Ccr4-Not complex. Journal of molecular biology 303: 131–143. 11023781 10.1006/jmbi.2000.4131

[41] Farago M, Nahari T, Hammel C, Cole CN, Choder M (2003) Rpb4p, a subunit of RNA polymerase II, mediates mRNA export during stress. Molecular biology of the cell 14: 2744–2755. 12857861 10.1091/mbc.E02-11-0740 PMC165673

[42] Lotan R, Goler-Baron V, Duek L, Haimovich G, Choder M (2007) The Rpb7p subunit of yeast RNA polymerase II plays roles in the two major cytoplasmic mRNA decay mechanisms. The Journal of cell biology 178: 1133–1143. 17875743 10.1083/jcb.200701165 PMC2064649

[43] Chen X, Ruggiero C, Li S (2007) Yeast Rpb9 plays an important role in ubiquitylation and degradation of Rpb1 in response to UV-induced DNA damage. Molecular and cellular biology 27: 4617–4625. 17452455 10.1128/MCB.00404-07 PMC1951484

[44] Azzouz N, Panasenko OO, Deluen C, Hsieh J, Theiler G, et al (2009) Specific roles for the Ccr4-Not complex subunits in expression of the genome. RNA 15: 377–383. 19155328 10.1261/rna.1348209 PMC2657018

[45] Reese JC (2013) The control of elongation by the yeast Ccr4-not complex. Biochimica et biophysica acta 1829: 127–133. 22975735 10.1016/j.bbagrm.2012.09.001 PMC3545033

[46] Oberholzer U, Collart MA (1998) Characterization of NOT5 that encodes a new component of the Not protein complex. Gene 207: 61–69. 9511744 10.1016/s0378-1119(97)00605-7

[47] Woychik NA, Young RA (1989) RNA polymerase II subunit RPB4 is essential for high- and low-temperature yeast cell growth. Molecular and cellular biology 9: 2854–2859. 2674672 10.1128/mcb.9.7.2854-2859.1989 PMC362751

[48] Choder M (2004) Rpb4 and Rpb7: subunits of RNA polymerase II and beyond. Trends in biochemical sciences 29: 674–681. 15544954 10.1016/j.tibs.2004.10.007

[49] Sampath V, Sadhale P (2005) Rpb4 and Rpb7: a sub-complex integral to multi-subunit RNA polymerases performs a multitude of functions. IUBMB life 57: 93–102. 16036568 10.1080/15216540500078905

[50] Collart MA (2013) The NOT4 RING E3 ligase: a relevant player in co-translational quality control. ISRN Molecular Biology 2013: 548359. 10.1155/2013/548359 PMC4890865 27335678

[51] Nano N, Houry WA (2013) Chaperone-like activity of the AAA+ proteins Rvb1 and Rvb2 in the assembly of various complexes. Philosophical transactions of the Royal Society of London Series B, Biological sciences 368: 20110399. 23530256 10.1098/rstb.2011.0399 PMC3638392

[52] Jansen G, Wu C, Schade B, Thomas DY, Whiteway M (2005) Drag&Drop cloning in yeast. Gene 344: 43–51. 15656971 10.1016/j.gene.2004.10.016

[53] Panasenko O, Landrieux E, Feuermann M, Finka A, Paquet N, et al (2006) The yeast Ccr4-Not complex controls ubiquitination of the nascent-associated polypeptide (NAC-EGD) complex. The Journal of biological chemistry 281: 31389–31398. 16926149 10.1074/jbc.M604986200

[54] Collart MA, Struhl K (1994) NOT1(CDC39), NOT2(CDC36), NOT3, and NOT4 encode a global-negative regulator of transcription that differentially affects TATA-element utilization. Genes & development 8: 525–537. 7926748 10.1101/gad.8.5.525

[55] Pfaffl MW (2001) A new mathematical model for relative quantification in real-time RT-PCR. Nucleic acids research 29: e45. 11328886 10.1093/nar/29.9.e45 PMC55695

