# Supplementary figures and images for: The Not5 Subunit of the Ccr4-Not Complex Connects Transcription and Translation
Source: PLoS Genet. 2014 Oct 23;10(10):e1004569. doi: 10.1371/journal.pgen.1004569 (PMC4207488; doi:10.1371/journal.pgen.1004569)

# A

## Y2H Rpb4 interactions (Rpb4 bait)

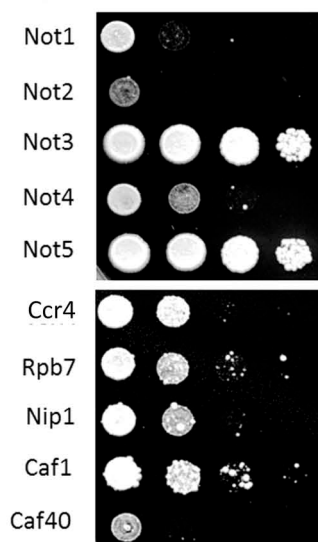

# D

## Y2H Nip1 interactions (Nip1 bait)

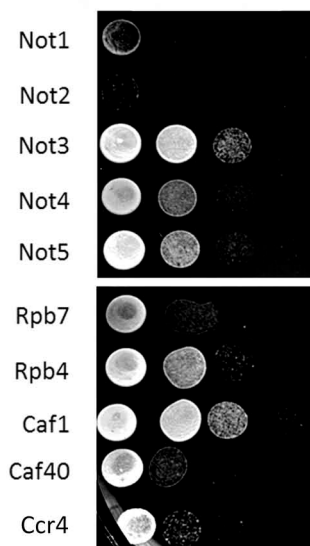

# B

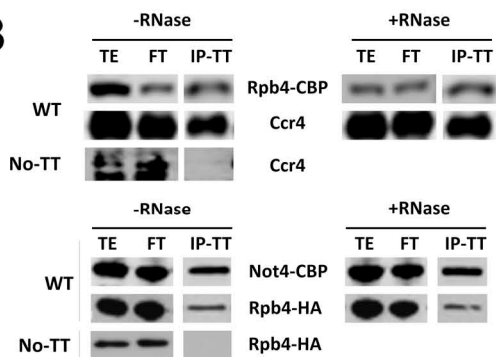

# C

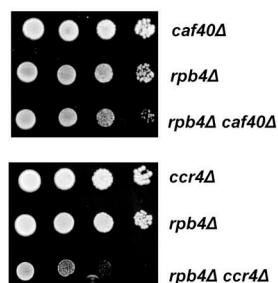

# E

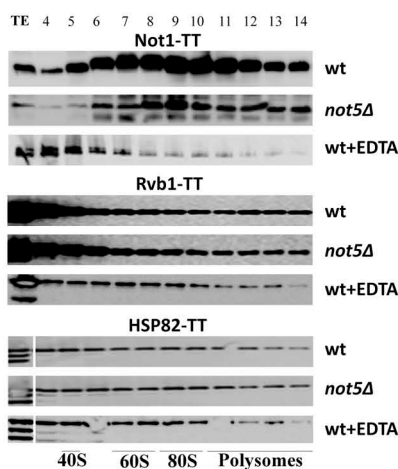

Supplement: Figure S1 — A. Serial dilutions of exponentially growing reporter cells expressing LexA-Rpb4 as a bait, and the indicated proteins fused to B42 as preys, were spotted on medium selective for the plasmids and indicative of an interaction between bait and prey. B. Upper panels: Rpb4-TT was immunoprecipitated from cells expressing Rpb4-TT and the presence of Rpb4 and Ccr4 was evaluated by western blotting with antibodies against CBP and Ccr4 respectively. A strain lacking any Tap-tagged protein was used as a control (No-TT). Immunoprecipitation with RNase-treated samples are also shown. Lower panels: Not4-TT was immunoprecipitated from cells expressing HA-tagged Rpb4 and Not4-TT. The presence of Not4 and Rpb4 in the total extract (TE), flow through (FT) and immunoprecipitate (IP-TT) was analyzed by western blotting with antibodies against CBP and HA respectively. A strain expressing HA-tagged Rpb4 but no Tap-tagged protein was used as a control (No-TT). Immunoprecipitation with RNase treated samples are also shown. C. Serial dilutions of exponentially growing cells from the indicated strains were spotted on plates and left to grow for several days at 30°C. D. Serial dilutions of exponentially growing reporter cells expressing LexA-Nip1 as a bait, and the indicated proteins fused to B42 as preys, were spotted either on medium selective for the plasmids and indicative of an interaction between bait and prey. E. Fractions from 7–47% sucrose gradients of extracts treated or not with EDTA as indicated from wild-type or not5Δ expressing Tap-tagged Not1, Rvb1 and Hsp82 were precipitated with TCA and analyzed by western blotting with PAP antibodies. The positions of 40S, 60S, 80S and polysomes are indicated under the blots. The numbers of the gradient fractions tested or the total extract (TE) are indicated at the top. The strains used that are not included in our strain list are MY4856 (Isogenic to BY4741 except MATα not1::NOT1-Taptag-URA3), MY5277 (MATa leu2Δ20 ura3Δ met15Δ his3Δ 1 ccr4 [file pgen.1004569.s001.pdf]

Merge

$\alpha$ -Myc  
*not5 $\Delta$*  + Myc-Not5

DAPI

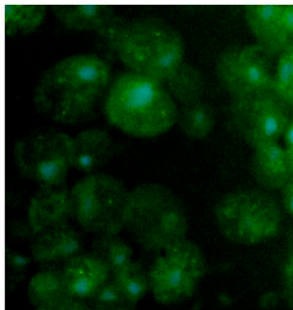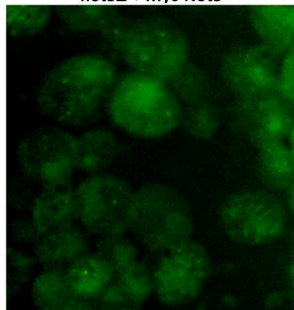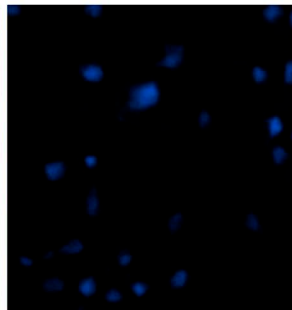

*not5 $\Delta$*  + Myc-Not5-NES

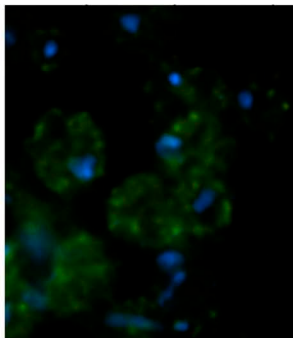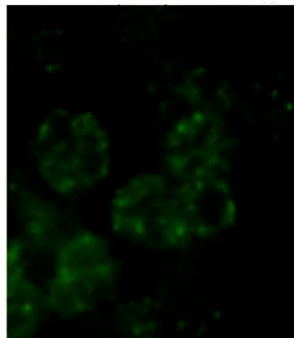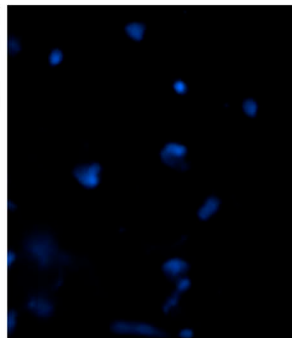

Supplement: Figure S2 — not5Δ cells expressing Rpb4-TT and the indicated Not5 derivatives, were grown exponentially and stained with anti-Myc antibodies (middle panels), or DAPI (right panels) or the pictures were merged (left panels). (PDF) [file pgen.1004569.s002.pdf]

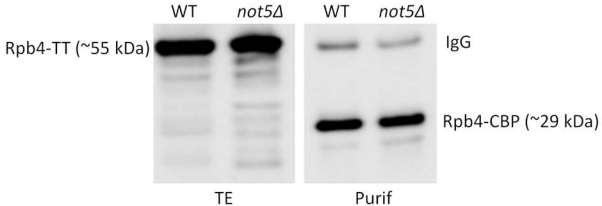

Supplement: Figure S3 — Rpb4 levels are the same in wt and not5Δ cells and Rpb4 immunoprecipitated to similar extent from both strains. TE: total extracts, Purif: Tap tag purification followed by TEV cleavage. (PDF) [file pgen.1004569.s003.pdf]

Nip1 mRNA

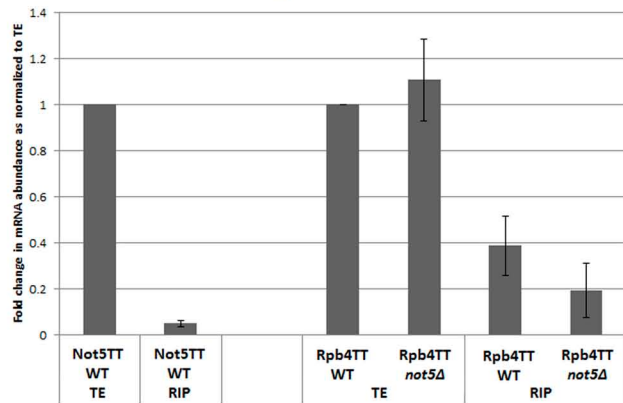

Rpb1 mRNA

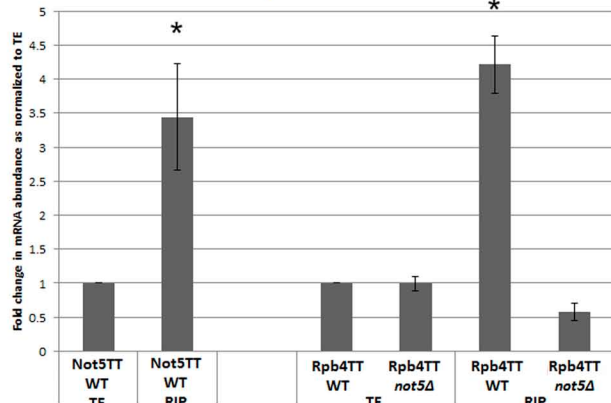

Nip1 mRNA

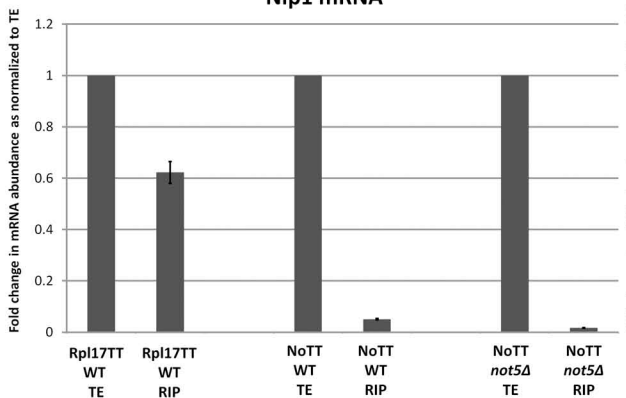

Rpb1 mRNA

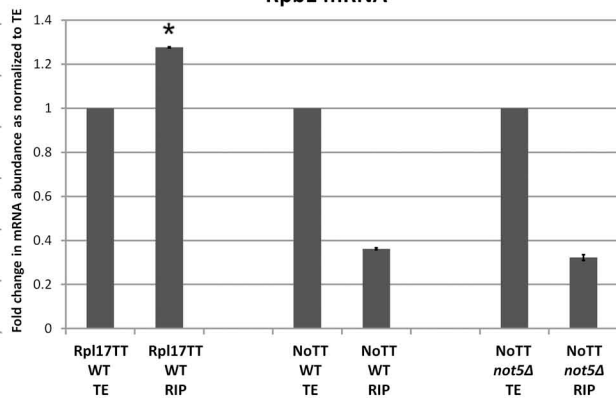

Supplement: Figure S4 — Rpb4 and Not5 are associated with RPB1 mRNA. The indicated Tap-tagged proteins were immunoprecipitated from total extracts of wild-type or not5Δ cells and RNA was purified from total extracts (TE) and the immunoprecipitates (RIP). The levels of RPB1 or NIP1 mRNA in 0.5 µg of TE and RIP RNA were measured by real-time PCR and expressed relative to the amount of these mRNAs identified in the TE of the wild-type (expressed as 1). * represents statistically significant enrichment of the RNA in the RIP relative to the TE at p<0.05. The strains used absent in our main strain list are MY5321 (Isogenic to BY4741 except not5::NOT5-TapTag-KanMX4) and MY9632 (MATa ade2 arg4 leu2,3112 trp1-289 ura3-52 rpl17b::RPL17B-Taptag-URA3; from Euroscarf). (PDF) [file pgen.1004569.s004.pdf]

-Leu, +Gal

Rpb4 plex

Not3

Not5

Rpb7

Nip1

Rpb7 plex

Not3

Not5

Rpb4

Nip1

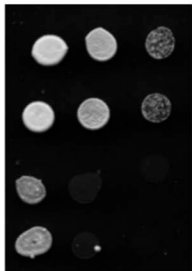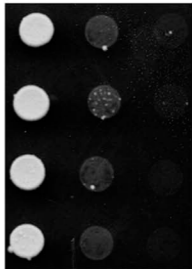

Supplement: Figure S5 — Rpb7 interacts with Not3 and Not5 to a lesser extent than Rpb4. 10-fold serial dilutions of exponentially growing reporter cells expressing LexA-Rpb4 or LexA-Rpb7 as a bait, and the indicated proteins fused to B42 as preys, were spotted on medium selective for the plasmids and indicative of an interaction between bait and prey. Note that Rpb7 interaction with Not3 or Not5 is weaker than that of Rpb4 if compared to known interaction with Nip1. (PDF) [file pgen.1004569.s005.pdf]

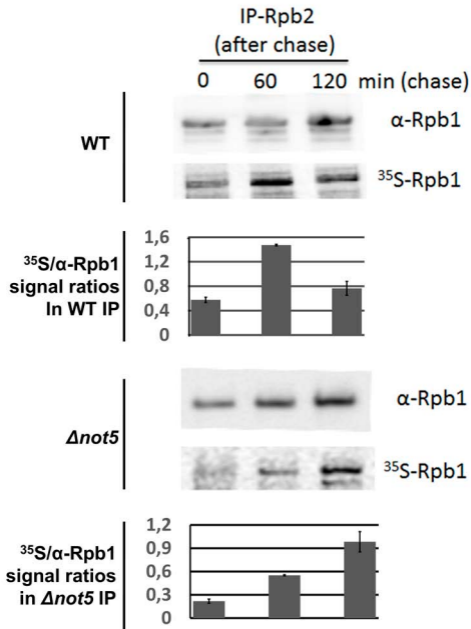

Supplement: Figure S7 — Delayed association of newly produced Rpb1 with Rpb2 in not5Δ. Exponentially growing wild-type and not5Δ cells expressing Rpb2-TT were pulse-labeled for 5 min with 35S-methionine (Met). We adjusted the input extracts to obtain similar levels of purified labeled proteins from both strains. We collected samples for Rpb2-TT right after the 5 min pulse, or 60 or 120 min after the chase. Rpb2 was purified by immunoaffinity followed by TEV cleavage. Eluates from the different time points were analyzed by western blotting with antibodies to Rpb1 (α-Rpb1) or with Phosphoimager (35S-Rpb1) to visualize the radioactive signal. Quantified ratios of the signal of 35S-Rpb1 relative to the signal of α-Rpb1 from the western blot are shown below the blots. (PDF) [file pgen.1004569.s007.pdf]

WT

*not5Δ*

0

2

4

8

0

2

4

8

CHX (hours)

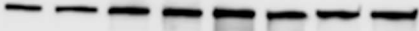

Rpb1 (~250 kDa)

Supplement: Figure S8 — Rpb1 levels are stable even after 8 hours of protein synthesis arrest both in wt and not5Δ cells. Cells were grown exponentially to an OD600 of 1.0 and then CHX (100 µg ml−1) was added. 0.8 OD600 units of wild-type cells and 1.6 OD600 units of not5Δ were collected at the indicated times after protein synthesis arrest. Total proteins prepared by post-alcaline lysis were analyzed by western blotting with antibodies against Rpb1. (PDF) [file pgen.1004569.s008.pdf]

## Nip1 mRNA in TE

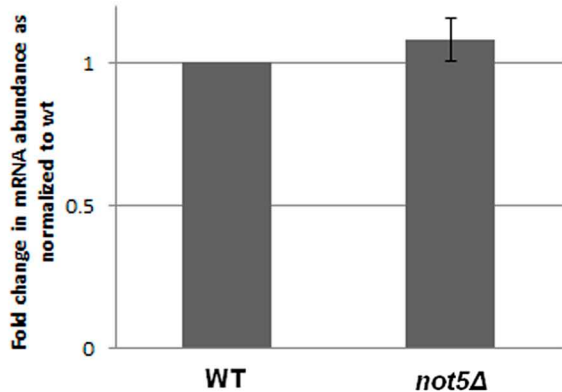

## Nip1 mRNA in polysomes

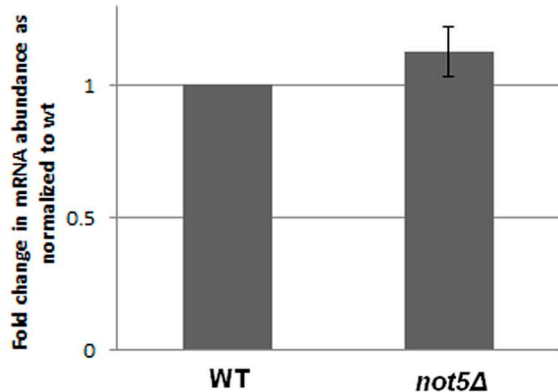

Supplement: Figure S9 — Similar NIP1 mRNA levels in wild-type and not5Δ. Total extracts from wild-type or not5Δ were separated on sucrose gradients as in Fig. 1C, and RNA was extracted from the total extracts (TE, left panel) or polysome fraction 14 (Fig. S15) (Polysomes, right panel). The amount of NIP1 mRNA was evaluated by RT followed by qPCR in 1 µg of total and polysomal RNA. The experiment was repeated 4 times and revealed no statistical significant difference of NIP1 mRNA levels in TE or polysomes of the wild-type compared to not5Δ. (PDF) [file pgen.1004569.s009.pdf]

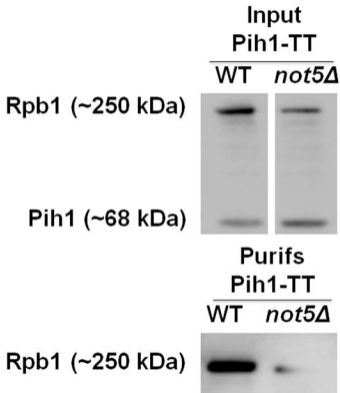

Supplement: Figure S10 — Less Rpb1 co-purifies with Pih1 from not5Δ than from wild type cells. The same experiment as in Figure 6B was performed with cells expressing Tap-tagged Pih1. (PDF) [file pgen.1004569.s010.pdf]

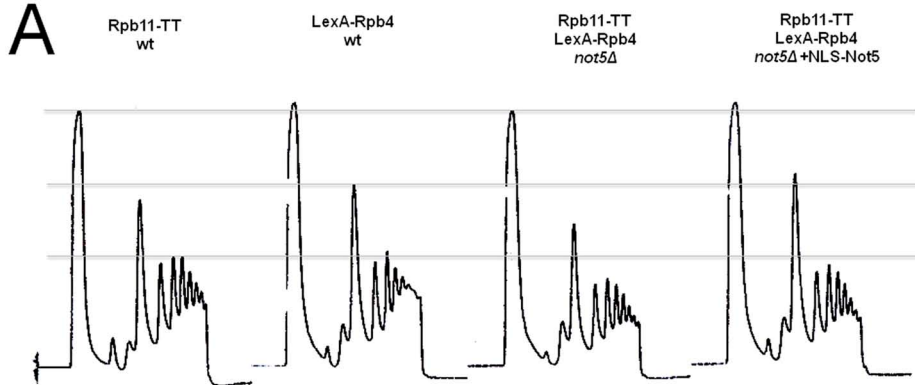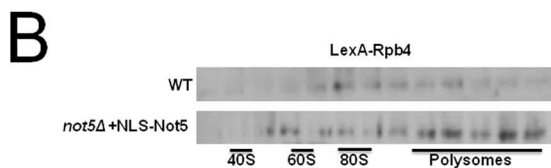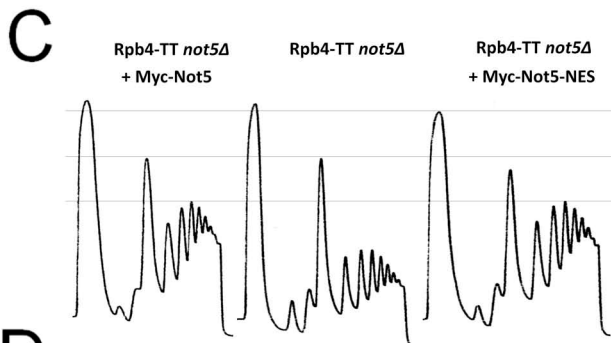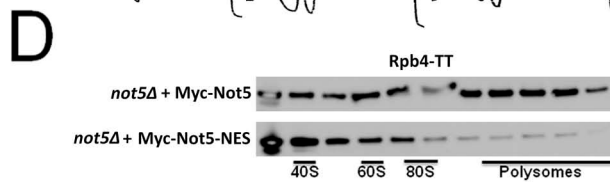

Supplement: Figure S11 — A. The total extracts from the 4 strains presented in Fig. 6E, left panel were separated on sucrose gradients and the polysome profiles are presented. B. Proteins in the different fractions of the wild-type and not5Δ +NLS-Not5 strains expressing LexA-Rpb4 were TCA precipitated and analyzed by western blotting to reveal the presence of LexA-Rpb4. C. The total extracts from the 3 strains presented in Fig. 6E, right panel were separated on sucrose gradients and the polysome profiles are presented. D. Proteins in the different fractions of the not5Δ + Myc-Not5 or not5Δ + Myc-Not5-NES strains expressing Rpb4-TT were TCA precipitated and analyzed by western blotting to reveal the presence of Rpb4-TT. (PDF) [file pgen.1004569.s011.pdf]

### Nip1 mRNA

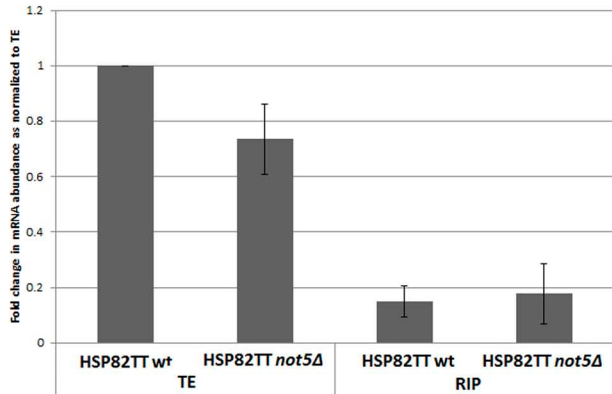

### Rpb1 mRNA

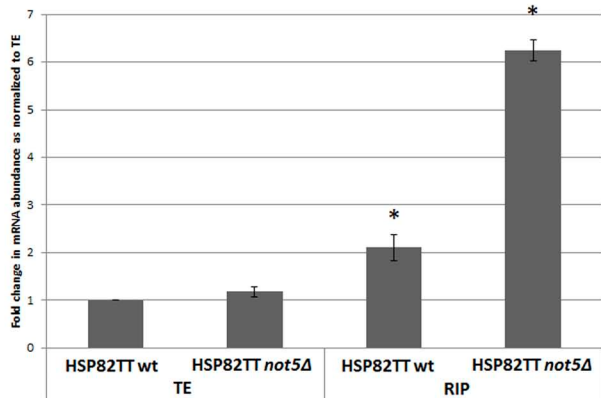

Supplement: Figure S12 — Hsp82 association with RPB1 mRNA is increased in cells lacking Not5. Tap-tagged Hsp82 was immunoprecipitated from total extracts of wild-type or not5Δ cells and RNA was purified from total extracts (TE) and the immunoprecipitates (RIP). The levels of RPB1 or NIP1 mRNA in 0.5 µg of TE and RIP RNA were measured by real-time PCR and expressed relative to the amount of these mRNAs identified in the TE of the wild-type (expressed as 1). * represents statistically significant enrichment of the RNA in the RIP relative to the TE at p<0.05. (PDF) [file pgen.1004569.s012.pdf]

*Rpb2*<sup>-/-</sup>

*CNot3*<sup>-/-</sup>

*Rpb2*<sup>-/-</sup>; *CNot3*<sup>-/-</sup>

α-Rpb1

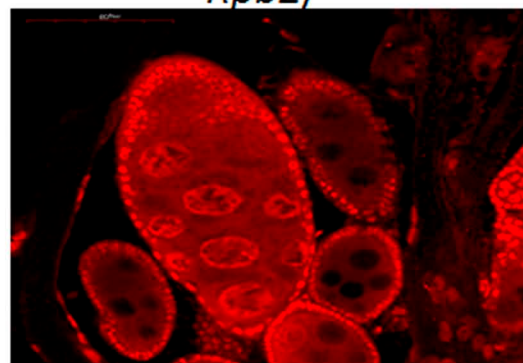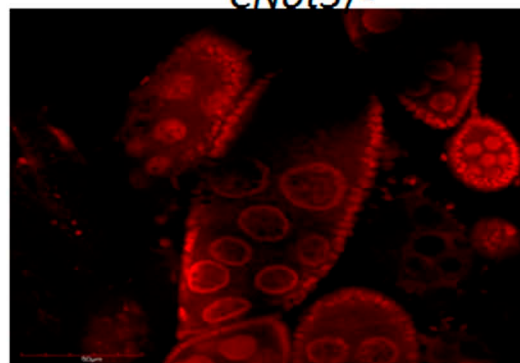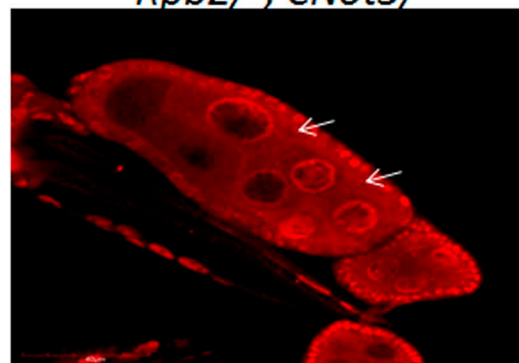

DAPI

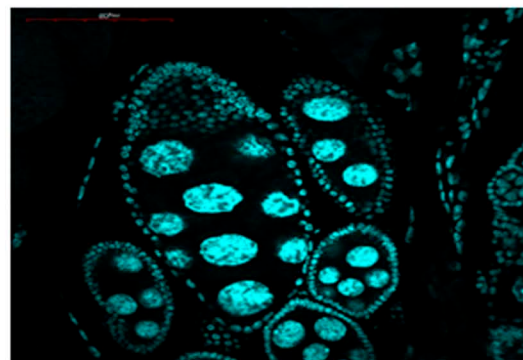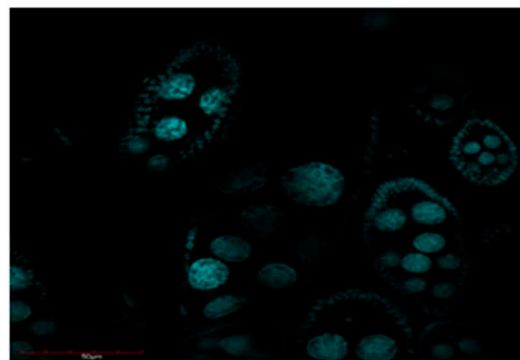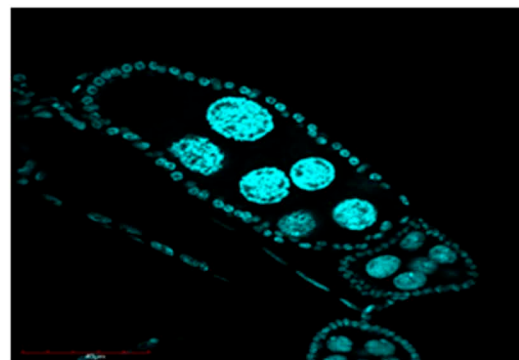

Merge

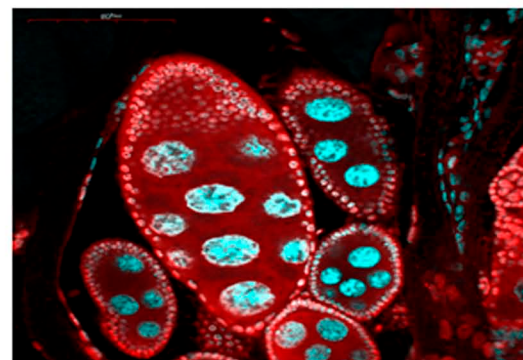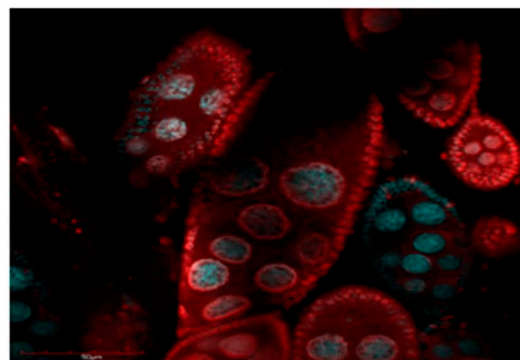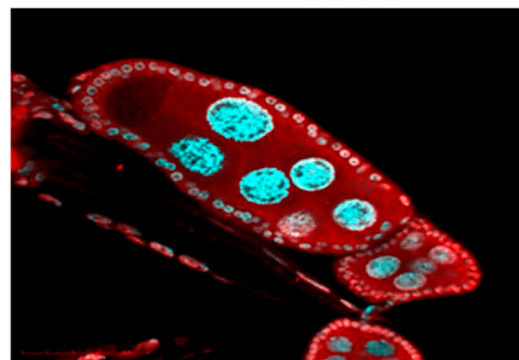

Supplement: Figure S13 — Egg chambers of different developmental stages ranging from stage 4 to stage 10 of Drosophila melanogaster of the indicated genotypes were stained with antibodies against Rpb1 or with DAPI, and the images were merged, as indicated. (PDF) [file pgen.1004569.s013.pdf]

**lwr1  
complexes**

**Npa3  
complexes**

**WT**

***not5Δ***

**WT**

***not5Δ***

**720—**

**480—**

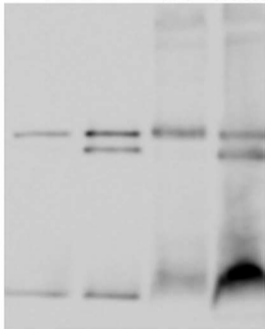

Supplement: Figure S14 — Total extracts from wild-type or not5Δ cells expressing the indicated Tap-tagged (TT) proteins were separated on native gels and analyzed by western blotting with anti-CBP antibodies. The strains 10269 (MATa ade2 arg4 leu2,3112 trp1-289 ura3-52 iwr1::IWR1-Taptag-URA3) and 10427 (MATa iwr1::IWR1-Taptag-URA3 not5::LEU2) and MY10266 (MATa ade2 arg4 leu2,3112 trp1-289 ura3-52 npa3::NPA3-Taptag-URA3) and MY10436 (MATa npa3::NPA3-Taptag-URA3 not5::LEU2) were used. (PDF) [file pgen.1004569.s014.pdf]

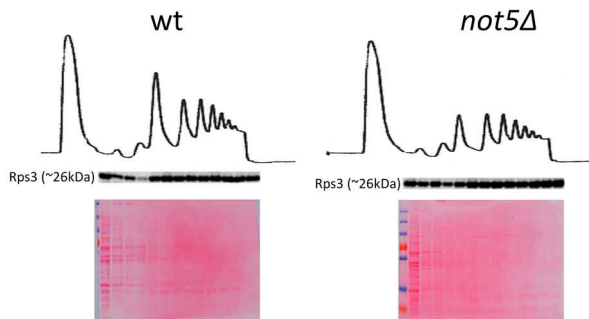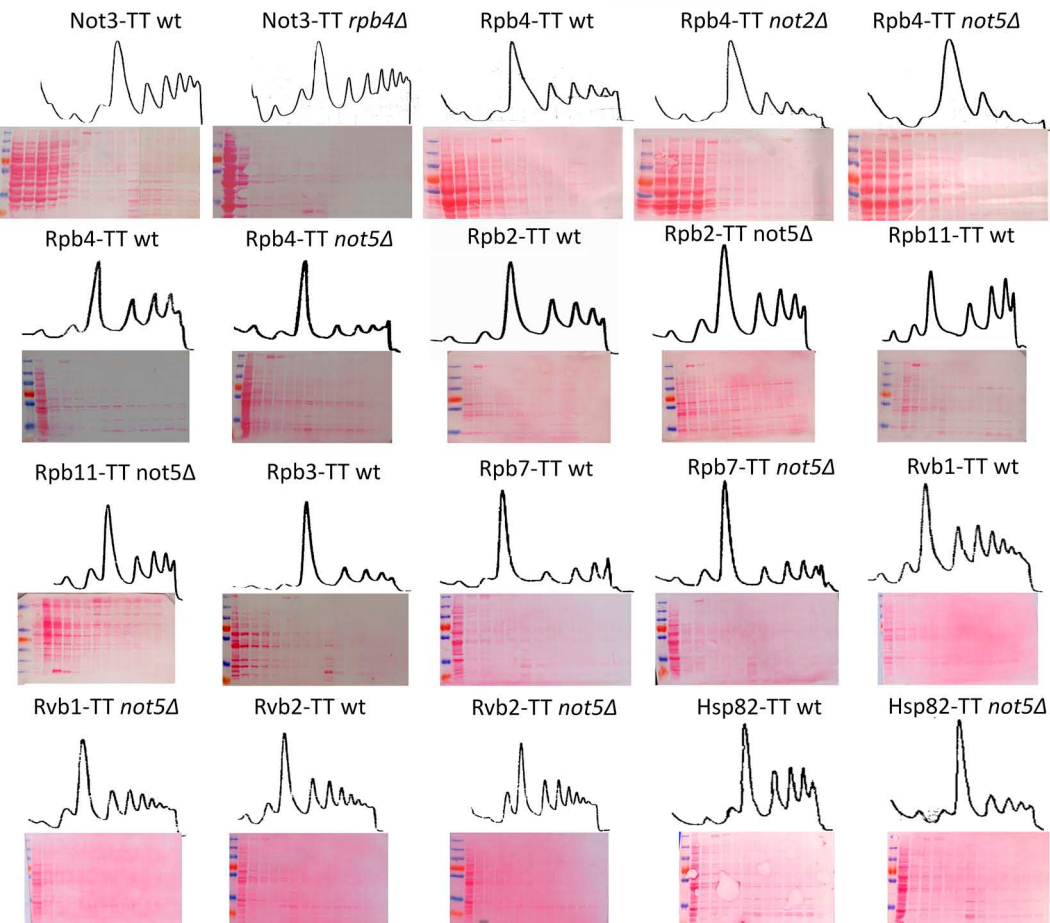

Supplement: Figure S15 — The different sucrose gradient fractionations shown in the manuscript are presented. On the top is presented a western-blot showing the distribution of Rps3, a ribosomal protein of the small ribosomal subunit, along a typical sucrose gradient of wt and not5Δ in this manuscript. (PDF) [file pgen.1004569.s015.pdf]

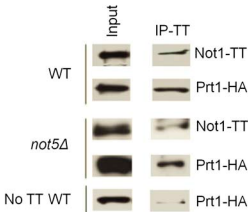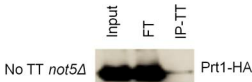

Supplement: Figure S16 — Prt1-HA is not immunoprecipitated non-specifically from WT or not5Δ cells. WT or not5Δ cells expressing Not1-TT or not expressing any Tap-tagged protein (No TT), and expressing Prt1-HA were incubated with IgG sepharose beads, washed and specifically bound proteins were eluted and analyzed by SDS-PAGE followed by western blotting with antibodies against CBP for the TT proteins or HA for Prt1-HA. (PDF) [file pgen.1004569.s016.pdf]
