# Supplementary material for: The Not5 Subunit of the Ccr4-Not Complex Connects Transcription and Translation
Source: PLoS Genet. 2014 Oct 23;10(10):e1004569. doi: 10.1371/journal.pgen.1004569 (PMC4207488; doi:10.1371/journal.pgen.1004569)
Supplement: Figure S6 — A lower molecular weight RNA Pol II complex lacking Rpb1 can be purified via several different RNA Pol II subunits. The indicated Tap-tagged RNA Pol II subunits were purified by a single step affinity purification and the purified proteins were analyzed on native gels and western blotting with anti-CBP antibodies (left panel) or anti-Rpb1 antibodies (right panel). The complex of a size compatible with mature RNA Pol II and a subcomplex enriched in not5Δ (*) are indicated. (PDF) [file pgen.1004569.s006.pdf]

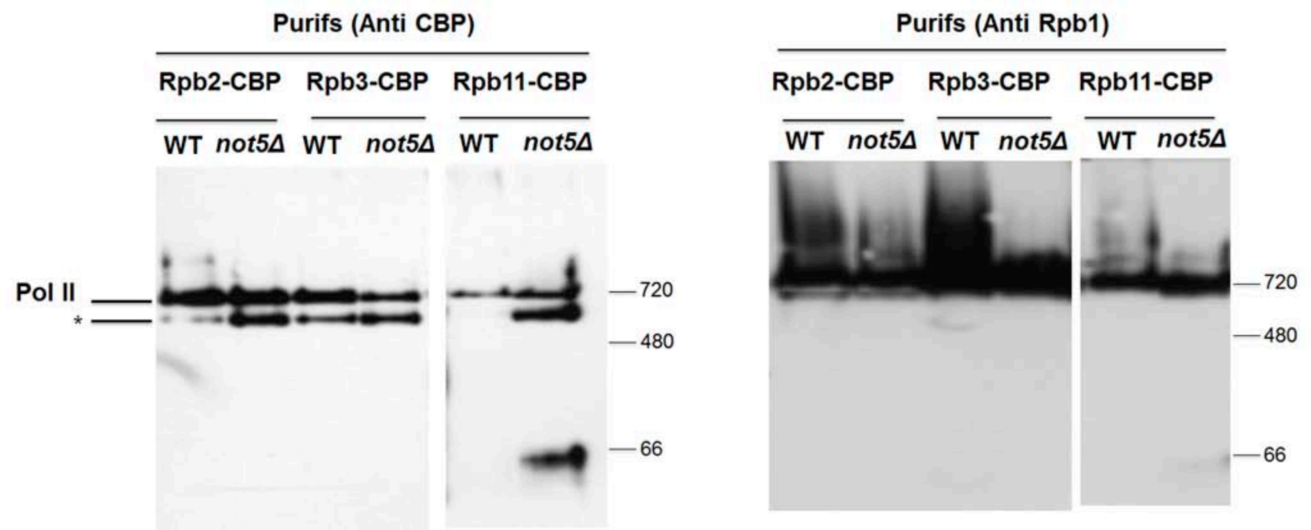

Purifs (Anti CBP)
Rpb2-CBP
Rpb3-CBP
Rpb11-CBP
WT
not5Δ
WT
not5Δ
WT
not5Δ
Pol II
*
720
480
66
Purifs (Anti Rpb1)
Rpb2-CBP
Rpb3-CBP
Rpb11-CBP
WT
not5Δ
WT
not5Δ
WT
not5Δ
720
480
66
