# Supplementary material for: The Not5 Subunit of the Ccr4-Not Complex Connects Transcription and Translation
Source: PLoS Genet. 2014 Oct 23;10(10):e1004569. doi: 10.1371/journal.pgen.1004569 (PMC4207488; doi:10.1371/journal.pgen.1004569)
Supplement: Table S1 — Rvb1 and Rvb2 are identified with various peptides in Not5 purification with LC/MS/MS. Tap-tagged Not5 was purified and the purified proteins were loaded on a native gel that was stained with Commassie. The entire lane was cut in slices and analyzed by LC/MS/MS. Table shows the polypeptides that identified Rvb1 and Rvb2 as co-purifying proteins with Not5. (DOCX) [file pgen.1004569.s017.docx]

| Protein name | Peptides identified in Not5 purifs |  |
| --- | --- | --- |
| RVB1 | VEGGFVGQIEAR |  |
|  | TALALAISQELGPK |  |
|  | LDPTIYESIQR |  |
|  | ALESNIAPVVVLASNR |  |
|  | GTEDVISPHGVPPDLIDR |  |
|  | TLPYDKDEIR |  |
|  |  |  |
| RVB2 | FVQCPEGELQK |  |
|  | TQGFLALFTGDTGEIR |  |
|  | AYLLFLDSAR |  |
|  | TTDMETIYELGNK |  |
