# Supplementary material for: The Not5 Subunit of the Ccr4-Not Complex Connects Transcription and Translation
Source: PLoS Genet. 2014 Oct 23;10(10):e1004569. doi: 10.1371/journal.pgen.1004569 (PMC4207488; doi:10.1371/journal.pgen.1004569)
Supplement: Table S2 — Yeast strains used in this work. (DOCX) [file pgen.1004569.s018.docx]

| **Strain** | **Genotype** | | **Source** |
| --- | --- | --- | --- |
| MY 290 | | *leu2::LexAOp6-LEU2 ura3 trp1 his3* | [[1](#_ENREF_1)] |
| MY2332 | | *MAT*a *leu2Δ0 ura3Δ0 met15Δ his3Δ1* | BY4741 |
| MY5615 | | Isogenic to BY4741 except *MAT*α *not4::HIS3MX4* | [[2](#_ENREF_2)] |
| MY5027 | | Isogenic to BY4741 except *not3::NOT3- Taptag -KANMX4* | [[2](#_ENREF_2)] |
| MY5460 | | *MAT*α *leu2 trp1 ade2-1 his3Δ200 rpb9::RPB9-TapTag-URA3* | Michel Werner |
| MY5461 | | *MAT*a *ade2 arg4 leu2-3,112 trp1-289 ura3-52* *rpb11::RPB11-TapTag-URA3* | Euroscarf |
| MY5462 | | *MAT*a *ade2 arg4 leu2-3,112 trp1-289 ura3-52* *rpb2::RPB2-TapTag-URA3* | Euroscarf |
| MY5463 | | *MAT*a *leu2 ura3-52 lys2 trp1 his3Δ200* *rpb7::RPB7-TapTag-URA3* | Michel Werner |
| MY5464 | | *MAT*α *leu2-3,112 ura3-52 his3Δ200 lys2* *rpb3::RPB3-TapTag-URA3* | Michel Werner |
| MY5675 | | *MAT*a *leu2Δ20 ura3Δ met15Δ his3Δ1 lys2Δ0 not5::NATMX4* | This work |
| MY6213 | | *MAT*α *gcn4Δ ura3-52 trp1Δ1 leu2::PET56 gal2 rpl25A::RPL25A-3HA-KanMX* | This work |
| MY8538 | | *MAT*α *rpb9::RPB9-Taptag-URA3 not5::NATMX4* | This work |
| MY8766 | | *MAT*a *leu2Δ0 ura3Δ0 met15Δ0 his3Δ1 rpb4::KanMX4* | Euroscarf |
| MY8856 | | *MAT*α *leu2Δ20 ura3Δ met15Δ lys2Δ0 his3Δ1* *not1::NOT1-Taptag-URA3 prt1::PRT1-HA3-KanMX4* | This work |
| MY8971 | | Isogenic to BY4741 except *rpb4::RPB4-Taptag-KanMX4* | This work |
| MY9021 | | *MAT*a *prt1::PRT1-HA3-KanMX4 hisΔ1* | This work |
| MY9027 | | *MAT*a *prt1::PRT1-HA3-KanMX4 not5::NATMX4* *his3 not1::NOT1-Taptag-URA3* | This work |
| MY9082 | | *rpb4::KanMX4 not4::HIS3MX4* | This work |
| MY9185 | | Isogenic to BY4741 except *MAT*α *rpb4::KanMX4 not3::NOT3-TapTag-KanMX4 lys2Δ0* | This work |
| MY9362 | | *MAT*α *rpb4::RPB4-Taptag-KanMX4 not5::NATMX4* | This work |
| MY9381 | | *MAT*α *prt1::PRT1-HA3-KanMX4 not5::NATMX4* *rpb4::RPB4-Taptag-KanMX4* | This work |
| MY9446 | | *MAT*a *rpb4::RPB4-Taptag-KanMX4 prt1::PRT1-HA3-KanMX4* | This work |
| MY9508 | | *MAT*a *rpb4::RPB4-Taptag-KanMX4 not2::NATMX4* | This work |
| MY9551 | | *MAT*a *not5::NATMX4 his3 ade2* *rpb2::RPB2-Taptag-URA3* | This work |
| MY9599 | | *rpb7::RPB7-Taptag-URA3 not5::NATMX4* | This work |
| MY9580 | | *MAT*a *rpb11::RPB11-Taptag-URA3 not5::NATMX4* | This work |
| MY9758 | | *MAT*a *ade2 arg4 leu2,3112 trp1-289 ura3-52 rvb1::RVB1-Taptag-URA3* | Euroscarf |
| MY9759 | | *MAT*a *ade2 arg4 leu2,3112 trp1-289 ura3-52 rvb2::RVB2-Taptag-URA3* | Euroscarf |
| MY10081 | | *MAT*α *gcn4Δ ura3-52 trp1Δ1 leu2::PET56 gal2* *rpb2::RPB2-Taptag-URA3 rpl25A::RPL25A-3HA-KanMX* | This work |
| MY10027 | | *MAT*a *rvb1::RVB1-Taptag-URA3 not5::NATMX4* | This work |
| MY10038 | | *MAT*a *rvb2::RVB2-Taptag-URA3 not5::NATMX4* | This work |
| MY10060 | | *MAT*a *ade2 arg4 leu2-3,112 trp1-289 ura3-52* *hsp82::HSP82TT-URA3* | Euroscarf |
| MY10349 | | *MAT*a *ade2 arg4 leu2-3,112 trp1-289 ura3-52* *hsp82::HSP82TT-URA3 not5::NATMX4* | This work |
| MY10065 | | *rpb9::RPB9-Taptag-URA3 rpb4:NATMX4* | This work |

**References**

1. Zervos AS, Gyuris J, Brent R (1993) Mxi1, a protein that specifically interacts with Max to bind Myc-Max recognition sites. Cell 72: 223-232.

2. Azzouz N, Panasenko OO, Deluen C, Hsieh J, Theiler G, et al. (2009) Specific roles for the Ccr4-Not complex subunits in expression of the genome. RNA 15: 377-383.
